# *GNAS* Mutations in Pseudohypoparathyroidism Type 1a and Related Disorders

**DOI:** 10.1002/humu.22696

**Published:** 2014-09-13

**Authors:** Manuel C Lemos, Rajesh V Thakker

**Affiliations:** 1 CICS-UBI, Health Sciences Research Centre, Faculty of Health Sciences, University of Beira InteriorCovilhã 6200-506, Portugal; 2 Academic Endocrine Unit, Nuffield Department of Clinical Medicine, University of Oxford, Oxford Centre for Diabetes, Endocrinology and Metabolism (OCDEM), Churchill HospitalHeadington, Oxford, OX3 7LJ, United Kingdom

**Keywords:** pseudohypoparathyroidism, pseudopseudohypoparathyroidism, Albright hereditary osteodystrophy, progressive osseous heteroplasia, Gs-alpha, GNAS

## Abstract

Pseudohypoparathyroidism type 1a (PHP1a) is characterized by hypocalcaemia and hyperphosphatemia due to parathyroid hormone resistance, in association with the features of Albright's hereditary osteodystrophy (AHO). PHP1a is caused by maternally inherited inactivating mutations of Gs-alpha, which is encoded by a complex imprinted locus termed *GNAS*. Paternally inherited mutations can lead either to pseudopseudohypoparathyroidism (PPHP) characterized by AHO alone, or to progressive osseous heteroplasia (POH), characterized by severe heterotopic ossification. The clinical aspects and molecular genetics of PHP1a and its related disorders are reviewed together with the 343 kindreds with Gs-alpha germline mutations reported so far in the literature. These 343 (176 different) mutations are scattered throughout the 13 exons that encode Gs-alpha and consist of 44.9% frameshift, 28.0% missense, 14.0% nonsense, and 9.0% splice-site mutations, 3.2% in-frame deletions or insertions, and 0.9% whole or partial gene deletions. Frameshift and other highly disruptive mutations were more frequent in the reported 37 POH kindreds than in PHP1a/PPHP kindreds (97.3% vs. 68.7%, *P* < 0.0001). This mutation update and respective genotype–phenotype data may be of use for diagnostic and research purposes and contribute to a better understanding of these complex disorders.

## Introduction

**Figure 1:**
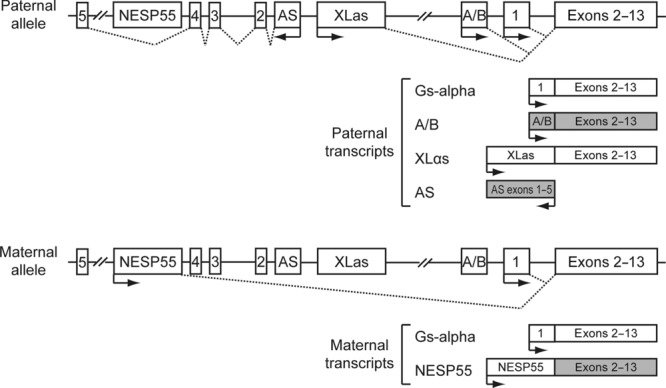
*GNAS* genomic structure and encoded transcripts. Gs-alpha is encoded by exons 1–13. Other transcripts produced by using alternative first exons that splice on to exons 2–13 are A/B (noncoding), XLαs, and NESP55. An AS noncoding transcript is also produced in the opposite direction using distinct exons. Gs-alpha is transcribed from both the paternal and maternal allele, except in selected tissues, such as renal proximal tubules, thyroid, gonads, and pituitary, in which expression occurs only from the maternal allele. A/B, XLαs, and AS transcripts are paternally expressed, and NESP55 transcripts are maternally expressed, as their promoters are located within DMRs (not shown). Exons are represented by boxes. Arrows indicate the direction of transcription of the different paternal and maternal transcripts. Dashed lines join exons that are spliced to produce the different transcripts. Shaded boxes represent noncoding transcripts.

Pseudohypoparathyroidism (PHP) is characterized by hypocalcaemia and hyperphosphataemia due to parathyroid hormone (PTH) resistance. This hormone resistance is usually caused by defects of the alpha subunit of the stimulatory form of the GTP-binding protein (Gs-alpha), which is a downstream signaling protein of the PTH receptor and of other G protein-coupled hormone receptors [Thakker, 2011]. Gs-alpha is encoded by the *GNAS* gene (MIM #139320), which is a complex imprinted locus that also produces additional coding and noncoding transcripts through the use of alternative promoters and alternative splicing, in a tissue-specific manner [Plagge et al., 2008; Turan et al., 2013] ( Fig. 1). Germline mutations of *GNAS* were initially discovered in 1990 [Patten et al., 1990] and have since been identified in many families with PHP. Inheritance in these is autosomal dominant with parental imprinting [Davies et al., 1993]. Several variants of PHP are recognized on the basis of their clinical, biochemical, and genetic features (Table 1).

**Table 1:** Clinical, Biochemical, and Genetic Features of Pseudohypoparathyroidism and Related Disorders

	PHP1a	PPHP	PHP1b	PHP1c	PHP2	POH	OC
AHO manifestations	Yes	Yes	No*	Yes	No	No*	No
Serum calcium	↓	N	↓	↓	↓	N	N
Serum phosphate	↑	N	↑	↑	↑	N	N
Serum PTH	↑	N	↑	↑	↑	N	N
Other hormonal resistance	Yes	No	No*	Yes	No	No	No
Heterotopic ossification	Yes (superficial)	Yes (superficial)	No	Yes (superficial)	No	Yes (deep tissues)	Yes (superficial)
Response to PTH:							
urinary cAMP	↓	↑	↓	↓	↑	↑	?
urinary phosphate	↓	↑	↓	↓	↓	↑	?
In vitro Gs-alpha activity	↓	↓	N	N	N	N or ↓	?
Inheritance	AD	AD	AD or Sporadic	AD	Sporadic	AD or Sporadic	AD or Sporadic
*GNAS* defect	Maternal inactivating mutations	Paternal inactivating mutations	Imprinting defects	Maternal inactivating mutations (rare)	None	Paternal inactivating mutations	Paternal inactivating mutations

* Except for some cases.

PHP, pseudohypoparathyroidism; PPHP, pseudopseudohypoparathyroidism; POH, progressive osseous heteroplasia; OC, osteoma cutis; AHO, Albright's hereditary osteodystrophy; PTH, parathyroid hormone; cAMP, cyclic adenosine monophosphate; GNAS, guanine nucleotide-binding protein, alpha-stimulating activity polypeptide; ↓, decreased; ↑, increased; N, normal; AD, autosomal dominant.

Patients with PHP type 1a (PHP1a) have PTH resistance (hypocalcaemia, hyperphosphataemia, elevated serum PTH, and a blunted increase in serum and urinary cyclic AMP [cAMP] and urinary phosphate following administration of PTH), in association with the features of Albright's hereditary osteodystrophy (AHO), which include short stature, obesity, subcutaneous calcifications, mental retardation, round facies, dental hypoplasia, and brachydactyly (i.e., shortening of the metacarpal bones, particularly the third, fourth, and fifth) [Thakker, 2011]. In addition to brachydactyly, other skeletal abnormalities of the long bones and shortening of the metatarsals may also occur. Patients with PHP1a may also show resistance to other hormones such as thyroid-stimulating hormone, follicle-stimulating hormone, luteinizing hormone, and growth hormone-releasing hormone, which also act via G protein-coupled receptors.

Patients with pseudopseudohypoparathyroidism (PPHP) exhibit most of the somatic features of AHO in the absence of PTH resistance. Both PHP1a and PPHP result from inherited inactivating mutations of *GNAS* and can coexist in the same family, but never in the same sibship. The development of one disorder or the other depends on the gender of the parent transmitting the genetic defect, as the hormone resistance is parentally imprinted [Turan et al., 2013]. Thus, PHP1a occurs in a child only when the mutation is inherited from a mother affected with either PHP1a or PPHP; and PPHP occurs in a child only when the mutation is inherited from a father affected with either PHP1a or PPHP.

PHP type 1b (PHP1b) is characterized by PTH resistance and the absence of somatic features of AHO in most patients. Patients with PHP1b do not have mutations within *GNAS* exons that encode Gs-alpha, but have methylation and imprinting defects resulting in the absence of expression of the maternal Gs-alpha [Turan et al., 2013].

Another related disorder is PHP1c, which is identical to PHP1a in terms of the presence of AHO and hormone resistance, but in contrast to PHP1a, in vitro assessment of Gs-alpha protein activity, using solubilized Gs-alpha from patient derived erythrocyte membranes, reveals no abnormality, and mutations of *GNAS* are usually not observed [Mantovani et al., 2006; Thiele et al., 2011].

Paternally inherited heterozygous inactivating mutations of Gs-alpha have also been identified in sporadic and familial cases of progressive osseous heteroplasia (POH) [Shore et al., 2002]. POH is characterized by dermal ossification during childhood, with progressive and extensive formation of heterotopic bone within deeper tissue, which often occurs along dermatomes [Cairns et al., 2013]. There is usually no hormone resistance or AHO features, and instead of the subcutaneous calcifications observed in AHO, it is the progression of dermal ossification into deep connective tissues that is the defining feature of POH [Shore et al., 2002]. Isolated osteoma cutis (OC) is also characterized by heterotopic ossification in the absence of hormone resistance or AHO features, but differs from POH in that the heterotopic ossification is limited to dermis and subcutaneous tissues. OC is often the initial manifestation of POH [Shore et al., 2010].

Finally, there is also a related disorder termed PHP type 2, which is characterized by hypocalcemia, PTH resistance, absence of AHO, and no *GNAS* mutations. In this disorder, there is a normal cAMP response to PTH, but no phosphaturic response. This indicates that the defect is distal to cAMP generation in the PTH-mediated signal transduction pathway, due to other molecular defects (e.g., mutations in *PRKAR1A* or *PDE4D*) [Linglart et al., 2011; Michot et al., 2012] or acquired causes such as vitamin D deficiency [Akin et al., 2010].

The aims of this study were to review all published Gs-alpha germline mutations and to investigate these for possible genotype–phenotype correlations.

## Gs-Alpha Germline Mutations

A database of published Gs-alpha germline mutations was constructed by searching the NCBI PubMed literature database for articles, using the keywords “mutation” combined with either “*GNAS*”, “Gs-alpha”, “Albright hereditary osteodystrophy”, “pseudohypoparathyroidism” or “progressive osseous heteroplasia”. Publications were analyzed for relevant content and a total of 93 articles presented results of mutation analysis with at least one identified Gs-alpha germline mutation. Articles were analyzed for evidence of duplication of data and kindreds that had been included in previous mutation studies were excluded from the analysis. Each published mutation was checked for accuracy by comparison to the *GNAS* wild-type sequence. Errors due to the incorrect assignment of nucleotide or codon numbers or translation errors between codon and amino acid residues were corrected whenever possible. Mutations shown only at the amino acid level were converted to single-nucleotide changes when it was possible to predict the altered base using the genetic code. When more than one nucleotide change could account for the amino acid change or when other ambiguous changes were indicated, the precise mutation was considered unavailable. The numbering of each nucleotide was changed, whenever necessary, to comply with current recommendations for mutation nomenclature [den Dunnen et al., 2001], whereby nucleotide +1 was the A of the ATG-translation initiation codon. Sequence variations were described in relation to the *GNAS* cDNA reference sequence (GenBank accession number NM_000516.4).

A total of 343 kindreds with heterozygous Gs-alpha germline mutations were identified in the literature and evaluated (Supp. Table S1). These 343 kindreds yielded a total of 176 different germline mutations. The 343 reported germline mutations were scattered across the entire Gs-alpha coding region and splice sites of the *GNAS* gene and consisted of 44.9% frameshift deletions or insertions, 28.0% missense mutations, 14.0% nonsense mutations, 9.0% splice-site mutations, 3.2% in-frame deletions or insertions, and 0.9% whole or partial gene deletions. A 4-bp deletion at codons 189–190 in exon 7 (c.565_568delGACT) was found to recur in 61 (17.8%) kindreds, thereby confirming a previously known mutational hot spot [Yu et al., 1995; Aldred et al., 2000; Elli et al., 2013b]. In addition to this mutational hot spot, mutations were also found to cluster in exon 1. A total of 61 (17.8%) kindreds had mutations in exon 1, and this overrepresentation was significant even after adjusting for the size of the exon (139 bp or 11.7% of the coding sequence) (chi-square goodness-of-fit test, *P* < 0.0001). The frequency of mutations at these sites did not differ among the phenotypic classes, with the exception of PHP1c, in which mutations were exclusively located in exon 13.

Among the reported 343 kindreds with Gs-alpha germline mutations, the most frequent phenotypes were PHP1a/ PPHP (297 kindreds) and POH (37 kindreds). Mutations in 25 POH kindreds were identical to those observed in PHP1a/PPHP. Whereas PHP1a is due to a maternally inherited mutation, both POH and PPHP are paternally inherited and it is remarkable that the same *GNAS* mutation causes POH in some families and PPHP in other families.

Less frequently reported phenotypes were PHP1c (five kindreds), PHP1b (one kindred), and isolated OC (three kindreds). The kindreds with PHP1c have mutations of residues at the carboxyl terminal of the protein (encoded by exon 13) that selectively affect receptor coupling but not adenylyl cyclase-activating functions of Gs-alpha [Thiele et al., 2011]. They are however unusual as the vast majority of cases of PHP1c do not have mutations of *GNAS* and are postulated to have defects elsewhere in the cAMP-dependent signaling pathway [Thiele et al., 2011]. The reported kindred with PHP1b [Wu et al., 2001] is also very unusual as this condition is generally caused by methylation and imprinting defects of the *GNAS* gene [Turan et al., 2013] and because the same mutation has been reported in a case of AHO [Ringel et al., 1996]. The three reported cases with isolated OC (i.e., superficial heterotopic ossification without AHO or hormone resistance) and a *GNAS* mutation were all infants (aged 1, 3, and 6 years) [Elli et al., 2013a; Huh et al., 2014], so it remains to be confirmed whether this is a true *GNAS*-associated phenotypic category or rather the first manifestation of POH.

Among the 343 reported kindreds, 39.3% were reported as familial, 25.4% were reported as sporadic *de novo*, and 35.3% had no information on inheritance. Sporadic *de novo* cases were more frequent in POH than in the other phenotypes (24.3% familial, 48.6% sporadic; 27.0% unknown; Fisher's exact test, *P* = 0.003), and this may reflect a reduced reproductive fitness associated with the severity of POH.

Although, for practical reasons, the term “germline mutation” is used throughout this article, one cannot exclude the possibility that some sporadic cases may be mosaics for a somatic mutation.

## Biological Relevance

### GNAS Locus and Transcripts

The *GNAS* gene (chromosome 20q13.3) is a complex imprinted locus that, in addition to Gs-alpha, also produces other coding and noncoding transcripts through the use of alternative promoters and alternative splicing [Plagge et al., 2008; Turan et al., 2013] ( Fig. 1).

Gs-alpha is encoded by exons 1–13, which span ∼20 kb [Kozasa et al., 1988]. Two expressed forms of Gs-alpha, of 52 and 45 kDa (designated long and short isoforms, respectively) are generated through alternative splicing of the 45-bp exon 3 that encodes 15 amino acids. In addition, the alternative use of a noncanonical TG 3′-splice site preceding exon 4 can result in the inclusion of an extra triplet coding for a serine residue after amino acids 87 and 72 of the long and short forms of Gs-alpha, respectively. Thus, two long and two short forms of Gs-alpha can be produced from the same precursor transcript, through tissue-dependent alternative splicing, and may have different regulatory properties [Kozasa et al., 1988; Novotny et al., 1998].

Gs-alpha is the alpha subunit of the stimulatory guanine nucleotide-binding protein, which belongs to the G protein family. This stimulatory heterotrimeric G protein (composed of an alpha, beta, and gamma subunit) is ubiquitously expressed and has a key role in the downstream signaling pathway of many hormones, through generation of the second messenger cAMP. In the basal, inactive state, this heterotrimeric G protein has guanosine diphosphate (GDP) bound to its alpha subunit. Ligand binding to the G protein-coupled receptor promotes GDP release and its replacement by GTP, which results in a switch to an active conformation and dissociation from the other subunits (beta and gamma), allowing the GTP-bound alpha subunit to activate its effectors. The alpha subunit has intrinsic GTPase activity that hydrolyses bound GTP to GDP, ensuring a turn-off mechanism that returns the system to the basal state [Weinstein et al., 2004].

Exons 2–13 can also be used with another three alternative first exons, located upstream, to produce additional transcripts. Exon extra-large αs (XLαs), located 35 kb upstream from exon 1, can splice onto exons 2–13 to produce XLαs. XLαs is a large variant of Gs-alpha that is expressed primarily in neuroendocrine tissues and the nervous system. The XLαs and Gs-alpha proteins are identical over their C-terminal portions, but they have distinct N-termini. XLαs is able to bind to beta and gamma subunits and mediate receptor-stimulated cAMP production, under certain circumstances, but the precise cellular actions of XLαs remain unknown [Bastepe, 2012].

The use of another first exon located 47 kb upstream of exon 1 produces NESP55 (neuroendocrine secretory protein of Mr 55,000), in which the coding region is restricted to its first exon and exons 2–13 constitute the 3′-untranslated region. NESP55 is expressed in neural tissues and belongs to the granin family, whose members are involved in endocrine and neuronal secretory pathways, and although the exact mechanisms of these proteins remain poorly understood, peptides derived from the granins have been shown to be involved in neuroendocrine, cardiovascular, endocrine, and inflammation systems [Bartolomucci et al., 2011].

Exon A/B, located 2.5 kb upstream from exon 1, can splice onto exons 2–13 to produce a noncoding RNA involved in the imprinting of the *GNAS* locus, although there is also evidence for an amino terminally truncated form of Gs-alpha that is derived from this transcript and may have inhibitory activity [Puzhko et al., 2011].

In addition to these four transcripts that use alternative first exons, the *GNAS* locus also encodes a noncoding transcript in the opposite direction, termed *GNAS* antisense (AS) transcript, which is also involved in the imprinting of the *GNAS* locus [Turan et al., 2013].

Gs-alpha transcripts are biallelically expressed except in a small number of tissues, including renal proximal tubules, thyroid, gonads, and pituitary, in which expression occurs only from the maternal allele. However, XLαs, A/B, and AS transcripts are paternally expressed, and NESP55 transcripts are maternally expressed. These encoded transcripts are parentally imprinted, as their promoters are located within differentially methylated regions (DMRs), and transcription from each promoter occurs exclusively from the nonmethylated allele [Linglart et al., 2013]. Imprinting of these DMR is under control of at least two imprinting control regions (ICRs) located within or close to the *GNAS* locus. One is located within the *syntaxin-16* (*STX16*) gene and controls the imprinting of the A/B DMR; the other is located in a region encompassing exons 3 and 4 of *GNAS* AS and controls the imprinting throughout the entire *GNAS* locus [Linglart et al., 2013].

### Functional Effects of Gs-Alpha Mutations

The most frequent germline Gs-alpha mutations are frameshift mutations that are expected to lead to a truncated protein or to nonsense-mediated decay [Kervestin et al., 2012]. Missense mutations are expected to be less disruptive and may provide insight into the functional domains of the protein and their interaction with other partners [Sunyaev, 2012]. Many of the Gs-alpha mutants are not expressed or do not localize to the cell membrane [Bastepe et al., 2005b]. Other mutants interfere with coupling to a variety of G protein-coupled receptors, and/or fail to activate adenylyl cyclase, leading to reduction or lack of cAMP generation [Turan et al., 2013]. Bioassays, using patients’ erythrocytes, platelets, and other tissues, reveal an approximate 50% reduction of Gs-alpha activity (measured by ligand-induced cAMP generation) (ranging between 40% and 75%), which is consistent with the heterozygous loss-of-function nature of these *GNAS* mutations [Ahrens et al., 2001; Mantovani, 2011].

The effects of the mutations are tissue-specific, depending on whether there is biallelic expression of Gs-alpha, or expression only from the maternal allele, due to imprinting of the paternal allele. Thus, in tissues such as the proximal renal tubules, thyroid, gonads, and pituitary, where the maternal allele is the predominant source of Gs-alpha, maternally inherited mutations will cause a marked reduction of Gs-alpha levels, leading to hormone resistance. In other tissues, where no parental imprinting occurs, there is a 50% reduction in Gs-alpha activity, sufficient to maintain a normal signaling activity in most cells, but leading to haploinsufficiency in others, such as those tissues involved in the AHO phenotype (e.g., growth plate) [Mantovani et al., 2004; Mantovani, 2011].

Missense mutations, leading to amino acid changes, appear to be underrepresented in exon 1, when compared with the rest of the coding sequence. Of 61 mutations described in exon 1, only six (9.8%) lead to an amino acid change, in contrast with 33.6% in exons 2–13 (Fisher's exact test, *P* = 0.001). A possible explanation for this observation is that missense mutations in exon 1 occur at the same rate as in other exons but are less likely to cause disease because the change of some amino acids in the amino terminus of the Gs-alpha may not significantly impair protein function [Thiele et al., 2010]. Alternatively, missense mutations in exon 1 may occur with reduced frequency because they may be very poorly tolerated.

Mutations that occur in exons 2–13 of Gs-alpha are expected to also affect other *GNAS* transcripts that share these exons. Unlike the A/B and NESP55 transcripts, for which these exons are noncoding, XLαs is expected to be affected by paternally inherited mutations. However, the role of these other mutated transcripts in human disease remains to be established. Loss of XLαs does not seem necessary for the development of these disorders, because mutations in exon 1, which is not used for XLαs, are sufficient for the disease phenotype. Nevertheless, recent studies have demonstrated an association between paternally inherited mutations in exons 2–13 (thus also affecting XLαs) and severe intrauterine growth retardation, feeding difficulties, and growth retardation during prime infancy [Lebrun et al., 2010; Richard et al., 2013], and these features are reminiscent of those observed in *Gnas*xl knockout mice [Plagge et al., 2004]. This indicates that mutations affecting other *GNAS* transcripts, in particular XLαs, can also produce significant effects.

In contrast with the germline mutations reported in the above described disorders, which are always inactivating mutations, somatic activating mutations of Gs-alpha have been identified in a variety of endocrine and nonendocrine tumors and also in individuals with the McCune–Albright syndrome [Weinstein et al., 2004]. These consist of point mutations that result in missense mutations in well-defined hotspots, usually within codons Arg201 or Gln227. These two amino acids are catalytically important for GTPase activity; therefore, these mutations cause constitutive activation by disrupting the turn-off mechanism of Gs-alpha [Weinstein et al., 2004]. There are no reported germline mutations in these codons, presumably because they are incompatible with embryonic development.

### Imprinting Defects of GNAS

*GNAS* imprinting defects have been identified in patients with familial and sporadic forms of PHP1b. In these patients, expression of maternal Gs-alpha is silenced in proximal renal tubules. As the maternal allele is the only source of Gs-alpha in the kidney (the paternal allele is normally silenced in this tissue), this maternal silencing results in marked reduction of Gs-alpha levels, leading to PTH resistance. The most consistent defect in these patients is loss of methylation at the exon A/B DMR in the maternal allele, leading to silencing of the downstream maternal Gs-alpha promoter [Izzi et al., 2012]. Structural mutations have been identified in ICRs that are important for the establishment and maintenance of the A/B and other DMRs [Izzi et al., 2012]. In particular, microdeletions of the *STX16* gene, located more than 200 kb upstream of the *GNAS* locus, have repeatedly been identified in familial cases of PHP1b, and are associated with loss of methylation at the A/B DMR. These include a recurrent 3 kb [Bastepe et al., 2003] and 4.4 Kb deletion [Linglart et al., 2005], and a more recently described 24.6 kb deletion [Elli et al., 2014b], all within the *STX16* gene. Other maternally inherited microdeletions have been identified within the NESP55 and/or *GNAS* AS regions [Bastepe et al., 2005a; Chillambhi et al., 2010; Richard et al., 2012], and these are associated with loss of methylation of other DMRs, in addition to the A/B DMR.

Most cases of PHP1b are sporadic and do not present deletions within the *GNAS* or *STX16* loci, despite showing broad loss of imprinting at the *GNAS* locus, including at the A/B DMR [Izzi et al., 2012]. A few cases have been shown to be due to paternal uniparental disomy involving whole or part of chromosome 20, encompassing the *GNAS* locus [Fernandez-Rebollo et al., 2010; Bastepe et al., 2011; Dixit et al., 2013]. In this situation, two normal copies of *GNAS* are both inherited from the father and therefore deficiency of Gs-alpha is predicted to occur in tissues in which expression occurs solely from the maternal allele. However, the genetic basis for most cases of sporadic PHP1b remains unknown.

Recently, imprinting defects in *GNAS* were also reported in a large proportion of patients with a PHP1a phenotype, in which no mutations were found in Gs-alpha coding exons [Mantovani et al., 2010; Elli et al., 2014a; Fernandez-Rebollo et al., 2013], suggesting that the clinical and genetic features of PHP1a and PHP1b may overlap.

### Animal Models

Both the human *GNAS* and the mouse ortholog *Gnas* genes have similar overall structure and imprinting patterns [Weinstein et al., 2004].

A knockout mouse model with disruption of *Gnas* exon 2 has been generated [Yu et al., 1998]. The homozygous *Gnas* knockout was shown to be embryonically lethal, with death before embryonic day 10.5, which would be expected for a ubiquitously expressed gene whose product is critical for many signaling pathways. The heterozygous knockout mice present phenotypes that depend on the parental origin of the mutation, thus confirming the imprinting-dependent expression of gene products. Both maternally (m−/+) and paternally (+/p−) inherited mutations result in significant early postnatal lethality. However, m−/+ newborns have higher birth weights, subcutaneous edema, developed neurological defects, with delayed development of the cerebellar cortex and thymic cortical atrophy, and immaturity of the kidneys. In contrast, +/p− newborns have lower birth weights and feeding problems. Surviving mice have delayed growth and development (both m−/+ and +/p−), but only the m−/+ adult mice exhibit PTH resistance, with elevation of PTH and decreased serum calcium levels. This is analogous to what is observed in humans, with maternal, but not paternal, transmission of AHO associated with PTH resistance. In addition, the study of expression of Gs-alpha in the m−/+ mice reveal almost complete lack of expression in the renal cortex, whereas +/p− mice have normal levels of expression in this tissue. However, in other tissues, there is only a 50% reduction in Gs-alpha expression, indicating that, at least in some tissues, the *Gnas* gene is also expressed from the paternal allele. Thus, it is likely that the tissue-specific effects of *Gnas* mutations will depend on whether the remaining 50% Gs-alpha activity is sufficient to maintain normal cellular functions.

Similar phenotypes were also observed in mice with a heterozygous missense mutation in exon 6, obtained through ethylnitrosourea mutagenesis experiments [Skinner et al., 2002].

Another mouse knockout model was developed by disrupting exon 1 [Chen et al., 2005; Germain-Lee et al., 2005], thereby targeting specifically the expression of Gs-alpha, but not the alternative *Gnas* gene products. These heterozygous knockout mice display a similar phenotype to the exon 2 disrupted mice, although they lacked the neurological abnormalities, suggesting that this feature in the exon 2 knockout could be due to the loss of Nesp55 that is highly expressed in the brain.

Although POH has not been reported in any of the existent mouse models, these heterozygous mice develop heterotopic ossification with increasing age [Huso et al., 2011; Cheeseman et al., 2012]. This phenotype is similar on both maternal and paternal inheritance, is confined to superficial tissues and does not affect deeper tissue (as in POH), and so resembles the subcutaneous ossification observed with AHO.

Other mouse models have been specifically designed to assess the roles of other *Gnas* transcripts. Disruption of exon XL ablates the XLαs transcript in mice with paternal inheritance of the disrupted XLαs allele and is associated with increased perinatal lethality, feeding problems, hypoglycaemia, growth retardation, reduced adiposity, increased basal metabolic rate, and increased sympathetic nervous system activity [Plagge et al., 2004; Xie et al., 2006]. Mice with disruption of exon 1A (equivalent to the human A/B exon) in the paternal allele have overexpression of Gs-alpha in renal proximal tubules and increased PTH sensitivity, indicating a role of the 1A region in the repression of Gs-alpha expression in some tissues [Williamson et al., 2004; Liu et al., 2005]. Mice with targeted deletion of Nesp55 have also been generated [Frohlich et al., 2010] and maternal inheritance of this deletion leads to loss of all maternal methylation imprints within the *Gnas* locus and to PTH resistance, thus providing a model of PHP1b. However, unlike human PHP1b patients, these mice show early postnatal lethality associated with hypoglycaemia. This unexpected phenotype has been attributed to loss of maternal XLαs imprinting, and XLαs overexpression, which can be reversed by simultaneous disruption of exon XL [Fernandez-Rebollo et al., 2012].

## Genotype–Phenotype Correlation

The reported Gs-alpha mutations are scattered throughout the entire coding region, with one mutational hotspot in exon 7 (c.565_568delGACT) accounting for 17.8% of cases. There appears to be no correlation between type or location of the Gs-alpha mutation, and onset of the disease, severity of endocrine resistances, or number of AHO signs in PHP1a/PPHP [Elli et al., 2013b].

As exons 2–13 are also shared by other *GNAS* transcripts, it is possible that the effects of the mutations are not limited to Gs-alpha. However, there is evidence that the phenotype caused by mutations in exon 1, which is unique to Gs-alpha, does not differ from the phenotype caused by mutations in exons 2–13 [Thiele et al., 2010]. Thus, this suggests that selective deficiency of Gs-alpha alone is sufficient to cause the hormonal and AHO manifestations, and cannot be compensated by other alternative transcripts.

There is a single reported mutation in exon 3 [Thiele et al., 2007]. The almost absence of mutations in exon 3 could be explained by the fact that splicing out this exon still produces a functional protein (short isoform of Gs-alpha). The reported mutation in exon 3 [Thiele et al., 2007] partially reduced Gs activity in the affected family members to about 70%–75% of normal (instead of the expected 50% reduction) and was associated with a somewhat milder phenotype, indicating a possible compensatory effect of the unaffected short Gs-alpha isoform.

**Figure 2:**
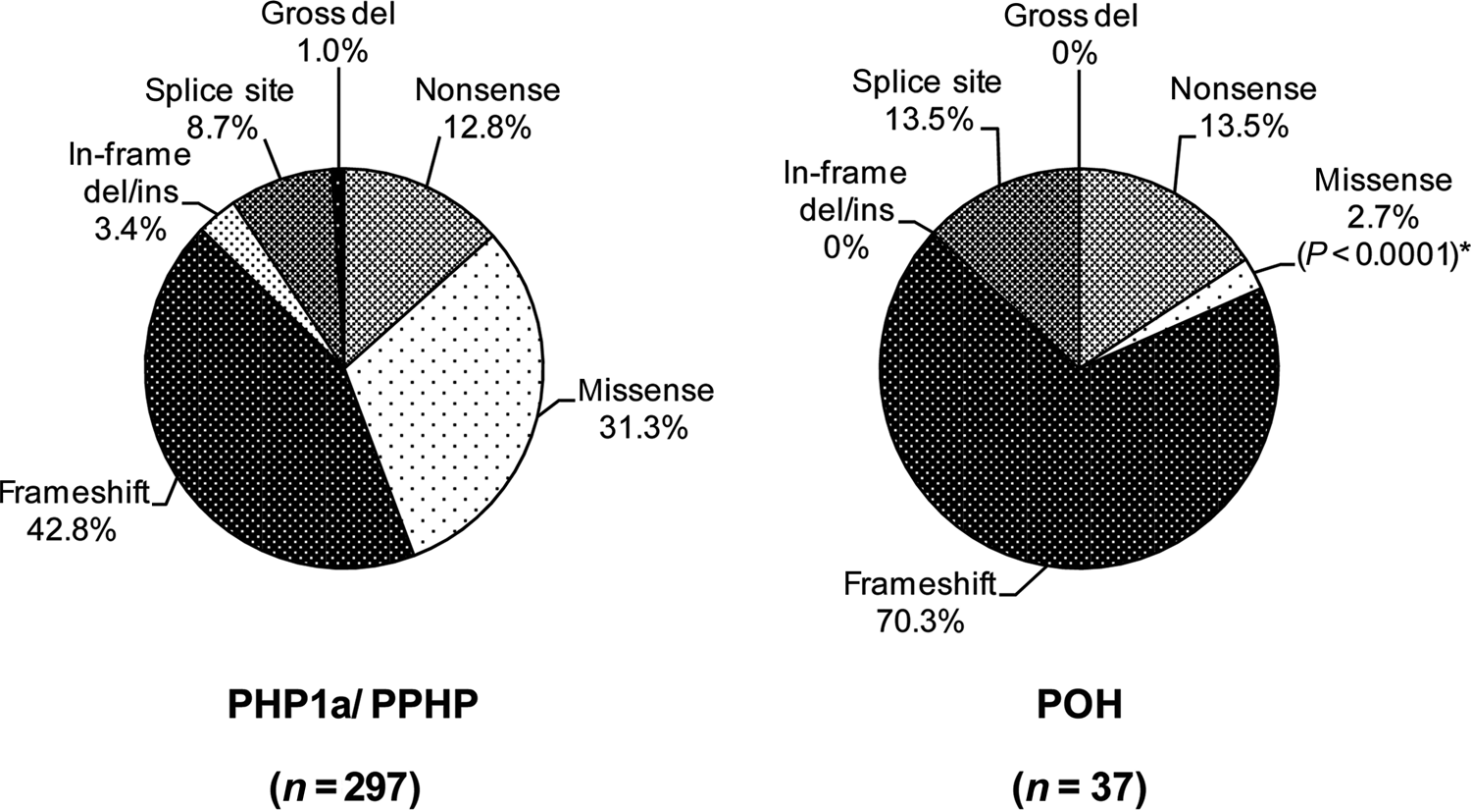
Frequencies of the types of *GNAS* mutations reported in 297 PHP1a/PPHP kindreds and 37 POH kindreds. Missense mutations were less frequent in POH than in PHP1a/PPHP kindreds (*Fisher's exact test, *P* < 0.0001). del, deletion; ins, insertion.

As POH and PPHP can be both caused by paternally inherited mutations of Gs-alpha, we compared the distribution of each mutation type between PHP1a/PPHP kindreds and POH kindreds and found that frameshift and other highly disruptive mutations, that are likely to cause null alleles, were significantly more frequent in POH than in PHP1a/PPHP kindreds, whereas the opposite was observed for missense mutations. This observation is consistent with the greater severity of the heterotopic ossifications in POH, as frameshift, nonsense, splice-site mutations, and gross deletions are more likely to severely disrupt RNA and protein expression than are missense mutations. Indeed, among 37 reported POH kindreds, only one (2.7%) was found to have a missense mutation [Chan et al., 2004], and this contrasts with PHP1a/PPHP in which missense mutations were present in 31.3% (Fisher's exact test, *P* < 0.0001) ( Fig. 2). This genotype–phenotype correlation has not been previously described. So far, only two other comprehensive mutation updates have been published. The first, published in 2000 [Aldred et al., 2000], involved only 52 reported kindreds, and was undertaken before POH was recognized as a *GNAS*-related disorder [Shore et al., 2002], impeding any analysis of genotype–phenotype correlation. Another more recent update identified a total of 192 different families (128 different *GNAS* mutations), but did not distinguish those with POH from those with PHP1a/PPHP [Elli et al., 2013b]. If the data were available, it would be interesting to see whether the severity of the more common subcutaneous ossifications, which occur in PHP1a and PPHP, is also influenced by the type of mutation.

As POH is exclusively paternally inherited, it has been suggested that the aggravated phenotype may be due to the simultaneous effect of disruption of XLαs, which shares the same coding region as Gs-alpha [Bastepe, 2012]. As XLαs, like Gs-alpha, activates cAMP signaling, it is possible that their combined loss, caused by a paternally inherited disruptive mutation, will result in a more severe phenotype. However, the existence of several POH kindreds with mutations in exon 1, which is not transcribed in XLαs, does not support this hypothesis.

These findings of different frequencies of mutation types in PHP1a/ PPHP and POH may contribute to the understanding of the variable expression of these disorders; however, it should be noted that several mutations are shared by both POH and PPHP patients and the mechanisms that determine the altered phenotypic expression of these mutations remain to be elucidated. A recent study by Cairns et al. (2013) presented an interesting hypothesis to explain the appearance of the POH lesions, which typically follow a dermomyotomal, and sometimes unilateral, distribution. By blocking Gs-alpha activity, through the use of a dominant negative mutant protein, in a subset of chick somites (the progenitors that give rise to dermis and muscle), they observed rapid ectopic cartilage and bone induction in a distribution corresponding to the injected somites. This suggests that somatic mutations in a progenitor cell of somitic origin, during embryogenesis, may act on a background of germline haploinsufficiency to cause loss of heterozygosity at the *GNAS* locus and result in a mosaic distribution of subcutaneous ectopic cartilage and bone formation in the progeny of these affected cells [Cairns et al., 2013]. This is also in agreement with previous studies in conditional knockout mice with biallelic inactivation of Gs alpha, which develop ectopic ossification in the subcutis and skeletal muscles [Castrop et al., 2007; Regard et al., 2013]. Perhaps this “second hit” mechanism is more likely to result in POH on a background of a severely disrupting mutation in Gs-alpha, thus explaining the overwhelming frequency of these mutations in patients with POH. However, this would not explain why POH patients generally lack other features of AHO which are caused by paternally inherited mutations.

Although POH and PPHP are clinically distinct disorders, a few patients with POH have also been reported to express some AHO-like features and even hormone resistance [Adegbite et al., 2008]. It has therefore been suggested that POH may represent an extreme phenotype of extra-skeletal ossification at the far end of the phenotypic spectrum of *GNAS*-based disorders [Adegbite et al., 2008].

## Clinical and Diagnostic Relevance

The clinical diagnosis of PHP1a is based on the presence of PTH resistance (i.e., hypocalcaemia, hyperphosphataemia, and elevated serum PTH) in association with the features of AHO, which includes short stature, obesity, subcutaneous calcification, mental retardation, round facies, and brachydactyly [Thakker, 2011]. Although rarely necessary, the demonstration of a blunted response of nephrogenous cAMP and phosphate secretion following exogenous PTH administration can contribute to the diagnosis [Todorova-Koteva et al., 2012]. Patients also frequently present resistance to other hormones such as thyroid-stimulating hormone, follicle-stimulating hormone, luteinizing hormone, and growth hormone-releasing hormone. Patients with PPHP exhibit most of the somatic features of AHO in the absence of PTH resistance, and their identification is often facilitated in the context of a family history of PHP1a.

Although the primary clinical finding of PHP1a is hypocalcaemia, which can lead to various neuromuscular defects including seizures, PHP1a patients frequently do not present with hypocalcaemia until after infancy. This delayed onset of PTH resistance may be explained by recent studies in knockout mice, which demonstrated that the manifestation of PTH resistance caused by the maternal loss of Gs-alpha occurs after early postnatal life, due to gradual development of paternal Gs-alpha silencing in renal proximal tubules [Turan et al., 2014].

Genetic testing, through DNA sequencing of the 13 Gs-alpha coding exons and intron–exon boundaries, will identify a mutation in most patients with PHP1a, PPHP, and POH, and rarely in PHP1c. However, it cannot distinguish between these related disorders or determine the parental origin of the mutation. In patients with PHP1b, epigenetic defects can be identified by methylation analysis of DMRs in the *GNAS* locus [Izzi et al., 2012]. The identification of a mutation in an index case allows a correct diagnosis and the possibility of predictive genetic testing in relatives, which can be excluded from further follow-up in the case of a negative result.

Conventional DNA sequencing will fail to reveal a mutation in some patients with a clinical diagnosis of PHP1a/PPHP. The proportion of PHP1a/PPHP cases without Gs-alpha mutations could not be determined in this database analysis because of the underlying bias to report chiefly on identified mutations. However, this proportion has been estimated to be approximately 20%–40%, based on some of the larger mutation analysis studies [Ahrens et al., 2001; Linglart et al., 2002; De Sanctis et al., 2003; Adegbite et al., 2008; Elli et al., 2013b]. Negative mutation results could be due to clinical phenocopies, or to mutations not easily detected by standard DNA sequence analysis, such as mutations in the promoter or untranslated regions, deep intronic mutations or gross gene deletions, or to mutations in other unknown genes. Recent studies have demonstrated that among cases diagnosed as PHP1a without mutations in Gs-alpha, 60% [Mantovani et al., 2010] to 80% [Fernandez-Rebollo et al., 2013] had imprinting defects of *GNAS*, suggesting the existence of a degree of overlap between molecular and clinical features of PHP1a and PHP1b and a need to review the current classification of these disorders.

Clinical heterogeneity makes genetic counseling a challenging task, especially in the case of paternal inheritance, because it can lead to either a mild expression of PPHP or a severe expression of POH [Lebrun et al., 2010]. Why paternal inheritance of a *GNAS* mutation should result in PPHP in some families and POH in others is unclear. The variable expressivity of phenotypes among patients with the same *GNAS*-inactivating mutation, even within the same family, suggests the influence of other modifier genes or environmental factors.

Treatment of PTH resistance is similar to that of other forms of hypoparathyroidism and consists of administration of active vitamin D metabolites (e.g., calcitriol) and oral calcium supplements, to maintain normal serum calcium levels. In patients with PHP1a or PHP1c, additional endocrine disorders, in particular hypothyroidism, hypogonadism, and growth hormone deficiency, should be screened for and corrected if necessary. There are no specific treatments for the various AHO features.

## Future Prospects

Analysis of patients with PHP1a and related disorders over the past several years has provided valuable information about the complexity of the *GNAS* locus. Although there has been an increasing amount of clinical and molecular data on these disorders, many aspects of their pathogenic mechanisms remain incompletely understood. Further identification of patients with novel *GNAS* mutations, along with their detailed clinical and biochemical characterization, may contribute to a better understanding of this complex locus.

The variable expression of phenotypes, even among patients with the same *GNAS* mutation, poses a challenging problem and suggests a role of other genetic loci, epigenetic modifications, or environmental factors. In particular, the question of why paternally inherited mutations can cause PPHP in some families and POH in others, needs to be addressed. The observation of a higher frequency of disruptive mutations in POH may contribute toward the explanation.

The majority of mutations of Gs-alpha simultaneously affect other *GNAS*-derived transcripts that share the same exons, but the role of these additional mutated transcripts in human disease, if any, remains to be clarified. The development of several knockout mouse models, targeting different transcripts of the gene, will continue to provide some insight into this issue.

Finally, clinical and molecular data have shown that there is a certain degree of overlap between the various subtypes of PHP, which adds yet another layer of complexity and will likely challenge the classical classification of these disorders.

## Supporting Information

Yes

Table S1. Germline mutations of the Gs-alpha encoding GNAS gene
