# Supplementary material for: GNAS Mutations in Pseudohypoparathyroidism Type 1a and Related Disorders
Source: Hum Mutat. 2014 Sep 13;36(1):11–9. doi: 10.1002/humu.22696 (PMC4309471; doi:10.1002/humu.22696)
Supplement: Supplementary file 1 — Table S1. Germline mutations of the Gs-alpha encoding GNAS gene [file humu0036-0011-sd1.pdf]

Supp. Table S1. Germline mutations of the Gs-alpha encoding *GNAS* gene

Mutation[a]	Predicted Effect on Protein	Mutation Type[b]	Phenotype[c]	Reference	Original designation
**Exon 1**					
c.1-10_2del	p.(?)	fs	PPHP	[Elli et al., 2013b]	c.1-10_2del
c.1A>G	p.(M1?)	ms	PHPIA, PPHP	[Patten et al., 1990]	codon 1 (ATG→GTG)
c.1A>G	p.(M1?)	ms	AHO	[Aldred et al., 2000b]	c.1A>G
c.1A>G	p.(M1?)	ms	PHPIA, PPHP	[Klaassens et al., 2010]	c.1A>G
c.2T>G	p.(M1?)	ms	PPHP	[Long et al., 2007]	c.2T>G
c.3G>A	p.(M1?)	ms	PPHP	[Puzhko et al., 2011]	c.3G>A
c.3G>A	p.(M1?)	ms	PHPIA	[Fernandez-Rebollo et al., 2013]	c.3G>A
c.7T>C	p.(Cys3Arg)	ms	PHPIA, PPHP	[Alves et al., 2013; Elli et al., 2013b]	Cys3→Arg
c.21dupT	p.(Lys8*)	fs	PHPIA	[Germain-Lee et al., 2003]	21insT
c.21dupT	p.(Lys8*)	fs	PHPIA, PPHP	[Long et al., 2007]	c.21insT
c.21dupT	p.(Lys8*)	fs	PHPIA	[Elli et al., 2013b]	c.21_22insT
c.34C>T	p.(Gln12*)	ns	POH	[Eddy et al., 2000]	Q12X (CAG→TAG)
c.34C>T	p.(Gln12*)	ns	PHPIA (2 kindreds)	[Fernandez-Rebollo et al., 2013]	c.34C>T
n/a (del6ins14)	n/a	fs	PHPIA	[Freson et al., 2008b]	codon 24-25 replaced by 14bp
c.77T>A	p.(Ile26Asn)	ms	PHPIA	[Long et al., 2007]	c.77T>A
c.83dupA	p.(Gln29Alafs*25)	fs	PHPIA	[De Sanctis et al., 2003]	c.83-84insA
c.85C>T	p.(Gln29*)	ns	PHPIA (2 kindreds)	[Germain-Lee et al., 2003]	85C>T
c.85C>T	p.(Gln29*)	ns	PHPIA, PPHP (3 kindreds)	[Long et al., 2007]	c.85C>T
c.85C>T	p.(Gln29*)	ns	PHPIA	[Nwosu et al., 2009]	nt85C>t
c.85C>T	p.(Gln29*)	ns	POH/ PHPIA	[Lebrun et al., 2010]	c.85C>T
c.85C>T	p.(Gln29*)	ns	POH (2 kindreds)	[Lebrun et al., 2010]	c.85C>T
c.85C>T	p.(Gln29*)	ns	PHPIA, PPHP (3 kindreds)	[Elli et al., 2013b]	c.85C>T
c.85C>T	p.(Gln29*)	ns	OC	[Elli et al., 2013a]	c.85C>T
c.85C>T	p.(Gln29*)	ns	PHPIA	[Fernandez-Rebollo et al., 2013]	c.85C>T
c.85C>T	p.(Gln29*)	ns	PHPIA	[Cho et al., 2013]	c.85C>T
c.85C>T	p.(Gln29*)	ns	PHPIA	[Wu, Y. L. et al., 2014]	c.85C>T
c.91C>T	p.(Gln31*)	ns	AHO	[Aldred et al., 2000b]	c.91C>T
c.91C>T	p.(Gln31*)	ns	PHPIA, PPHP	[De Sanctis et al., 2003]	c.91C>T
c.91C>T	p.(Gln31*)	ns	PHPIA (2 kindreds)	[Fernandez-Rebollo et al., 2013]	c.91C>T
c.93delG	p.(Lys32Argfs*26)	fs	PHPIA	[Thiele et al., 2010]	c.93delG
c.94A>T	p.(Lys32*)	ns	PHPIA, PPHP	[Park et al., 2010]	c.94A>T
c.94A>T	p.(Lys32*)	ns	PHPIA	[Cho et al., 2013]	c.94A>T
c.97G>A	p.(Asp33Asn)	ms	PHPIA	[Elli et al., 2013b]	c.97G>A
c.103C>T	p.(Gln35*)	ns	PHPIA	[Germain-Lee et al., 2003]	103C>T
c.103C>T	p.(Gln35*)	ns	PHPIA	[Long et al., 2007]	c.103C>T
c.103C>T	p.(Gln35*)	ns	PHPIA, PPHP	[Kacerovska et al., 2008]	c.103C>T
c.103C>T	p.(Gln35*)	ns	PHPIA	[Freson et al., 2008b]	Q35X
c.103C>T	p.(Gln35*)	ns	PHPIA, PPHP	[Klagge et al., 2010]	103C>T

Mutation[a]	Predicted Effect on Protein	Mutation Type[b]	Phenotype[c]	Reference	Original designation
c.103C>T	p.(Gln35*)	ns	PHPIA (3 kindreds)	[Elli et al., 2013b]	c.103C>T
c.103C>T	p.(Gln35*)	ns	PHPIA	[Wu, Y. L. et al., 2014]	c.103C>T
c.110delA	p.(Tyr37Serfs*21)	fs	PHPIA	[Elli et al., 2013b]	c.110del
c.111C>G	p.(Tyr37*)	ns	AHO	[Aldred et al., 2000b]	c.111C>G
c.112delC	p.(Arg38Glyfs*20)	fs	PHPIA, PPHP	[Mantovani et al., 2000]	codon 38, delC
c.112delC	p.(Arg38Glyfs*20)	fs	PHPIA	[Elli et al., 2013b]	c.112del
c.115delG	p.(Ala39Profs*19)	fs	AHO	[Aldred et al., 2000b]	c.115delG
c.119_139+17del38	p.(?)	sp	PHPIA, PPHP	[Fischer et al., 1998]	exon 1/ intron 1, del38
c.124C>A	p.(Arg42Ser)	ms	PHPIA	[Fernandez-Rebollo et al., 2013]	c.124C>A
c.124C>T	p.(Arg42Cys)	ms	AHO	[Aldred et al., 2000b]	c.124C>T
c.125G>T	p.(Arg42Leu)	ms	PHPIA	[Balavoine et al., 2008]	R42L
c.136_138dupCTG	p.(Leu46dup)	if	PHPIA	[Lim et al., 2002]	codon 47, insCTG
c.136_138dupCTG	p.(Leu46dup)	if	PHPIA	[Fernandez-Rebollo et al., 2013]	c.136_138dup
**Intron 1**					
c.139+1G>C	p.(?)	sp	PHPIA	[Alvarez et al., 2010]	139+1G>C
c.139+1G>C	p.(?)	sp	POH	[Lebrun et al., 2010]	c.139+1G>C
n/a	p.(?)	sp	PHPIA	[Elli et al., 2013b]	c.139+1T>A [d]
c.140-12_211inv84	p.(?)	sp	PHPIA	[Fernandez-Rebollo et al., 2008]	inversion nt 56904042-56904139
**Exon 2**					
c.144dupT	p.(Gly49Trpfs*5)	fs	AHO	[Aldred et al., 2000b]	c.144-145insT
c.150dupA	p.(Ser51Ilefs*3)	fs	PHPIA, PPHP	[Ahrens et al., 2001]	codon 51, insA(GAA TCT→GAAATC)
c.188_189delTG	p.(Leu63Profs*3)	fs	PHPIA, PPHP	Authors unpublished data [§]	
c.191A>T	p.(His64Leu)	ms	PHPIA	[Long et al., 2007]	c.191A>T
**Intron 2**					
c.212+3_+6delAAGT	p.(?)	sp	PHPIA	[De Sanctis et al., 2003]	IVS2 +3_6delAAGT
c.212+3_+6delAAGT	p.(?)	sp	POH	[Long et al., 2007]	c.212+3delAAGT
c.212+3_+6delAAGT	p.(?)	sp	PHPIA	[Elli et al., 2013b]	c.212+2_+6del
**Exon 3**					
c.254dupA	p.(Asp85Glufs*3)	fs	PHPIA	[Thiele et al., 2007]	codon 85, insA
**Intron 3**					
c.257+1G>A	p.(?)	sp	PHPIA	[Elli et al., 2013b]	c.257+1G>A
c.258-2A>G	p.(?)	sp	AHO	[Ringel et al., 1996]	A→G (intron 3 acceptor sp)
**Exon 4**					
c.265delG	p.(Ala89Glnfs*11)	fs	PHPIA	[Ishikawa et al., 2001]	codon 88, delG
c.269_272delCCAA	p.(Thr90Lysfs*9)	fs	PHPIA, PPHP	[Linglart et al., 2002]	codon 89/90, delCAAC
c.275T>A	p.(Val92Glu)	ms	AHO	[Aldred et al., 2000b]	c.275T>A
c.278_312+8del43	p.(?)	sp	AHO	[Luttikhuis et al., 1994]	exon4/ intron4 del43
c.292_294delAAC	p.(Asn98del)	if	AHO	[Weinstein et al., 2001]	Asn98del
c.292_294delAAC	p.(Asn98del)	if	PHPIA	[Long et al., 2007]	c.292-294del

Mutation[a]	Predicted Effect on Protein	Mutation Type[b]	Phenotype[c]	Reference	Original designation
c.296T>C	p.(Leu99Pro)	ms	AHO	[Miric et al., 1993]	Leu99Pro (T→C)
c.302_303delAG	p.(Glu101Glyfs*3)	fs	PHPIA, PPHP	[Yu, D. et al., 1999]	codon 100/101, delAG
c.305C>A	p.(Ala102Glu)	ms	PHPIA, PPHP	[Ahrens et al., 2001]	codon 102 (GCG→GAG)
c.305C>A	p.(Ala102Glu)	ms	PHPIA	[Pinsker et al., 2006]	c.305C>A
c.305C>A	p.(Ala102Glu)	ms	PHPIA	[Elli et al., 2013b]	c.308C>A
c.305C>T	p.(Ala102Val)	ms	PHPIA, PPHP	[Ahrens et al., 2001]	codon 102 (GCG→GTG)
c.308T>C	p.(Ile103Thr)	ms	AHO	[Aldred et al., 2000b]	c.308T>C
c.308T>C	p.(Ile103Thr)	ms	PHPIA	[Lim et al., 2002]	I103T
c.308T>C	p.(Ile103Thr)	ms	PHPIA	[Elli et al., 2013b]	c.311T>C
**Intron 4**					
c.312+5G>A	p.(?)	sp	PHPIA	[Lietman et al., 2008]	IVS4+5G→A
c.312+5G>A	p.(?)	sp	PHPIA	[Fernandez-Rebollo et al., 2013]	c.312+5G>A
c.313-11A>G	p.(?)	sp	PHPIA	[Rickard et al., 2003]	IVS4 -11A→G
**Exon 5**					
c.317T>C	p.(Ile106Thr)	ms	PHPIA	[Reis et al., 2010]	c.673T>C, I106T [d]
c.317T>G	p.(Ile106Ser)	ms	PHPIA	[Riepe et al., 2005]	c.364T>G, Ile106Ser (ATT→AGT) [d]
n/a (ins21)	n/a	if	PHPIA	[Freson et al., 2008b]	codon 110, ins 21 bp
c.330G>C	p.(Met110Ile)	ms	PHPIA	[Fernandez-Rebollo et al., 2013]	c.330G>C
c.343C>T	p.(Pro115Ser)	ms	PHPIA	[Ahmed et al., 1998]	codon 115 (CCC→TCC)
c.343C>T	p.(Pro115Ser)	ms	PHPIA	[Savas Erdeve et al., 2010]	Pro115Ser (CCC→TCC)
c.343_344insT	p.(Pro115Leufs*25)	fs	PHPIA	[Inta et al., 2014]	codon 115 CCC→CTCC
c.344C>T	p.(Pro115Leu)	ms	PHPIA, PPHP	[Ahrens et al., 2001]	codon 115 (CCC→CTC)
c.344C>T	p.(Pro115Leu)	ms	PHPIA, PPHP	[De Sanctis et al., 2003]	c.344C>T
c.344_345insT	p.(Val117Argfs*23)	fs	POH	[Shore et al., 2002]	344-345insT
c.344_345insT	p.(Val117Argfs*23)	fs	PHPIA	[Park et al., 2010]	c.344-345insT
c.344_345insT	p.(Val117Argfs*23)	fs	POH	[Lebrun et al., 2010]	c.345-346insT [d]
c.344_345insT	p.(Val117Argfs*23)	fs	PHPIA	[Elli et al., 2013b]	c.347_348insT
c.344_345insT	p.(Val117Argfs*23)	fs	PHPIA	[Cho et al., 2013]	c.344_345insT
c.348delC	p.(Val117Trpfs*16)	fs	PHPIA	[Shapira et al., 1996]	codon 116, delC
c.348delC	p.(Val117Trpfs*16)	fs	PHPIA	[Mantovani et al., 2000]	codon 116, delC
c.348delC	p.(Val117Trpfs*16)	fs	AHO	[Aldred et al., 2000b]	c.348delC
c.348delC	p.(Val117Trpfs*16)	fs	PHPIA	[Lim et al., 2002]	codon 115, delC
c.348delC	p.(Val117Trpfs*16)	fs	POH (2 kindreds)	[Shore et al., 2002]	348delC
c.348delC	p.(Val117Trpfs*16)	fs	PHPIA, PPHP	[De Sanctis et al., 2003]	c.348delC
c.348delC	p.(Val117Trpfs*16)	fs	PHPIA	[Fernandez-Rebollo et al., 2013]	c.348delC
c.348dupC	p.(Val117Argfs*23)	fs	PHPIA (2 kindreds)	[De Sanctis et al., 2003]	c.348-349insC
c.348dupC	p.(Val117Argfs*23)	fs	PHPIA (2 kindreds)	[Jin et al., 2011]	c.348-349insC
c.348dupC	p.(Val117Argfs*23)	fs	PHPIA (3 kindreds)	[Elli et al., 2013b]	c.351dup

Mutation[a]	Predicted Effect on Protein	Mutation Type[b]	Phenotype[c]	Reference	Original designation
c.348_349insT	p.(Val117Cysfs*23)	fs	PHPIA	[de Sanctis et al., 2004]	c.348-349insT
c.355delC	p.(Leu119Trpfs*14)	fs	PPHP	[Schimmel et al., 2010]	c.355delC
c.360delC	p.(Asn121Thrfs*12)	fs	PHPIA	[Elli et al., 2013b]	c.363del
c.366delC	p.(Glu123Argfs*10)	fs	PHPIA, PPHP	[De Sanctis et al., 2003]	c.366delC
c.395T>C	p.(Leu132Pro)	ms	AHO	[Aldred et al., 2000b]	c.395T>C
c.419_420delTT	p.(Phe140*)	fs	PHPIA	[Elli et al., 2013b]	c.421_422del
c.420dupT	p.(Asp141*)	fs	PHPIA, PPHP	[Ahrens et al., 2001]	codon 140, insT(TTT GAC→TTTTGA)
c.432delC	p.(Glu145Asnfs*27)	fs	PHPIA, PPHP	[Linglart et al., 2002]	del C432
**Intron 5**					
c.432+1G>A	p.(?)	sp	PHPIA, PPHP	[Wilson et al., 1994]	intron5 +1G→A
c.432+1G>A	p.(?)	sp	AHO	[Aldred et al., 2000b]	c.432+1G>A
c.432+1G>A	p.(?)	sp	PPHP	[Rickard et al., 2003]	c.432+1G>A
c.432+1G>A	p.(?)	sp	PHPIA	[Elli et al., 2013b]	c.435+1G>A
**Exon 6**					
c.457C>G	p.(Leu153Val)	ms	PPHP	[Cho et al., 2013]	c.457C>G
n/a	p.(Trp154*)	ns	PHPIA, PPHP	[Linglart et al., 2002]	W154X
c.466G>A	p.(Asp156Asn)	ms	PHPIA	[Linglart et al., 2002]	D156N (A→G)
c.470_472delAAG	p.(Glu157del)	if	PHPIA	[Long et al., 2007]	c.469-471del
c.472G>A	p.(Gly158Arg)	ms	PHPIA	[Fernandez-Rebollo et al., 2013]	c.472G>A
c.473dupG	p.(Val159Serfs*15)	fs	AHO	[Aldred et al., 2000b]	c.473-474insG
c.475G>A	p.(Val159Met)	ms	PHPIA	[Linglart et al., 2002]	V159M (A→G) [d]
c.475G>A	p.(Val159Met)	ms	PPHP	[Elli et al., 2013b]	c.478G>A
c.476T>C	p.(Val159Ala)	ms	PPHP	[Rump et al., 2011]	c.476T>C
c.478C>T	p.(Arg160Cys)	ms	AHO	[Aldred et al., 2000b]	c.478C>T
c.478C>T	p.(Arg160Cys)	ms	PHPIA	[Elli et al., 2013b]	c.481C>T
c.489C>G	p.(Tyr163*)	ns	AHO	[Aldred et al., 2000b]	c.489C>G
c.493C>T	p.(Arg165Cys)	ms	AHO	[Miric et al., 1993]	Arg165Cys (C→T)
c.493C>T	p.(Arg165Cys)	ms	PHPIA	[Ahrens et al., 2001]	codon 165 (CGC→TGC)
c.493C>T	p.(Arg165Cys)	ms	PHPIA	[Elli et al., 2013b]	c.496C>T
c.509_510dupAG	p.(Leu171Serfs*2)	fs	PHPIA	[Fernandez-Rebollo et al., 2013]	c.509_510dup
c.517G>A	p.(Asp173Asn)	ms	PHPIA	[Freson et al., 2008b]	D173N
c.518_521delACTG	p.(Asp173Valfs*11)	fs	PHPIA	[Fernandez-Rebollo et al., 2013]	c.518_521delACTG
c.522_523delTG	p.(Ala175Profs*14)	fs	PHPIA	[Elli et al., 2013b]	c.523_524del
**Intron 6**					
c.531-2A>G	p.(?)	sp	PHPIA, PPHP	[Ahrens et al., 2001]	I6/E7 (agCTTC→ggCTTC)
c.531-1G>C	p.(?)	sp	PHPIA	[Linglart et al., 2002]	G→C (intron 6 acceptor sp)
**Exon 7**					
c.546delC	p.(Ile182Metfs*3)	fs	POH/ PHPIA	[Gelfand et al., 2007]	546delC
c.551delT	p.(Val184Glyfs*20)	fs	OC	[Elli et al., 2013a]	c.554del
c.555delC	p.(Lys186Serfs*18)	fs	PHPIA, PPHP	[De Sanctis et al., 2003]	c.555delC
c.565_568delGACT	p.(Asp189Metfs*14)	fs	AHO	[Weinstein et al., 1992]	codon 189/190 delGACT

Mutation[a]	Predicted Effect on Protein	Mutation Type[b]	Phenotype[c]	Reference	Original designation
c.565_568delGACT	p.(Asp189Metfs*14)	fs	AHO (4 kindreds)	[Yu, S. et al., 1995]	codon 189/190 delGACT
c.565_568delGACT	p.(Asp189Metfs*14)	fs	PHPIA, PPHP	[Yokoyama et al., 1996]	codon 189/190 delGACT
c.565_568delGACT	p.(Asp189Metfs*14)	fs	PHPIA, PPHP (2 kindreds)	[Ahmed et al., 1998]	codon 189/190 delGACT
c.565_568delGACT	p.(Asp189Metfs*14)	fs	PHPIA, PPHP	[Nakamoto et al., 1998]	codon 189/190 delGACT
c.565_568delGACT	p.(Asp189Metfs*14)	fs	PHPIA, PPHP	[Walden et al., 1999]	exon7 4bp del
c.565_568delGACT	p.(Asp189Metfs*14)	fs	PHPIA	[Mantovani et al., 2000]	codon 189/190 del4
c.565_568delGACT	p.(Asp189Metfs*14)	fs	AHO	[Aldred et al., 2000b]	c.565-568delGACT
c.565_568delGACT	p.(Asp189Metfs*14)	fs	POH	[Yeh et al., 2000]	codon 189/190 delGACT
c.565_568delGACT	p.(Asp189Metfs*14)	fs	PHPIA, PPHP (6 kindreds)	[Ahrens et al., 2001]	codon 189/190 delGACT
c.565_568delGACT	p.(Asp189Metfs*14)	fs	PHPIA, PPHP (3 kindreds)	[Linglart et al., 2002]	codon 189/190 delGACT
c.565_568delGACT	p.(Asp189Metfs*14)	fs	PHPIA	[Lim et al., 2002]	codon 189/190 delGACT
c.565_568delGACT	p.(Asp189Metfs*14)	fs	POH (3 kindreds)	[Shore et al., 2002]	565-568delGACT
c.565_568delGACT	p.(Asp189Metfs*14)	fs	POH, AHO	[Shore et al., 2002]	565-568delGACT
c.565_568delGACT	p.(Asp189Metfs*14)	fs	PHPIA, PPHP (3 kindreds)	[De Sanctis et al., 2003]	c.565-568delGACT
c.565_568delGACT	p.(Asp189Metfs*14)	fs	PHPIA (3 kindreds)	[Germain-Lee et al., 2003]	565-568del
c.565_568delGACT	p.(Asp189Metfs*14)	fs	PHPIA, PPHP (9 kindreds)	[Long et al., 2007]	c.565-568del
c.565_568delGACT	p.(Asp189Metfs*14)	fs	PHPIA, PPHP	[Semiz et al., 2009]	codon 189/190 del4
c.565_568delGACT	p.(Asp189Metfs*14)	fs	PHPIA	[Lecumberri et al., 2009]	c.565-568delGACT
c.565_568delGACT	p.(Asp189Metfs*14)	fs	PHPIA	[Dekelbab et al., 2009]	Nt565delGACT
c.565_568delGACT	p.(Asp189Metfs*14)	fs	POH (2 kindreds)	[Lebrun et al., 2010]	c.565-568delGACT
c.565_568delGACT	p.(Asp189Metfs*14)	fs	PHPIA, PPHP (2 kindreds)	[Jin et al., 2011]	c.565-568del
c.565_568delGACT	p.(Asp189Metfs*14)	fs	PHPIA, PPHP (4 kindreds)	[Elli et al., 2013b]	c.568_571del
c.565_568delGACT	p.(Asp189Metfs*14)	fs	POH/ PHPIA [f]	[Elli et al., 2013b] [Elli et al., 2013a]	c.568_571del
c.565_568delGACT	p.(Asp189Metfs*14)	fs	POH (2 kindreds)	[Elli et al., 2013a]	c.568_571del
c.565_568delGACT	p.(Asp189Metfs*14)	fs	PHPIA (3 kindreds)	[Fernandez-Rebollo et al., 2013]	c.565_568delGACT
c.565_568delGACT	p.(Asp189Metfs*14)	fs	PHPIA	[Inta et al., 2014]	codon 189/190 delGACT
c.565_568delGACT	p.(Asp189Metfs*14)	fs	POH	[Schrander et al., 2014]	c.565_568delGACT
c.568T>G	p.(Tyr190Asp)	ms	AHO	[Ringel et al., 1996]	Tyr190Asp
c.569_570delAT	p.(Tyr190Cysfs*19)	fs	PHPIA	[Jin et al., 2011]	c.569-570del
c.569_570delAT	p.(Tyr190Cysfs*19)	fs	PHPIA	[Fernandez-Rebollo et al., 2013]	c.569_570delAT
c.572_573delTG	p.(Val191Alafs*18)	fs	POH	[Lebrun et al., 2010]	c.571-572delTG [d]
c.575C>T	p.(Pro192Leu)	ms	PHPIA, PPHP	[Long et al., 2007]	c.575C>T
n/a	n/a	fs	PHPIA, PPHP	[Mouallem et al., 2008]	c.575C>A + 576-577insC [g]
c.583C>T	p.(Gln195*)	ns	PHPIA	[Balavoine et al., 2008]	Q195X

Mutation[a]	Predicted Effect on Protein	Mutation Type[b]	Phenotype[c]	Reference	Original designation
**Intron 7**					
c.586-1G>A	p.(?)	sp	PHPIA	[Rickard et al., 2003]	c.586-1G>A
c.586-1G>T	p.(?)	sp	PHPIA	[Long et al., 2007]	c.586-1G>T
**Exon 8**					
c.586G>A	p.(Asp196Asn)	ms	PHPIA, PPHP	[Winter et al., 2011]	D196N
c.617_618delGA	p.(Gly206Aspfs*3)	fs	PHPIA, PPHP	[Yu, D. et al., 1999]	codon 206, delGA
c.617_618delGA	p.(Gly206Aspfs*3)	fs	PHPIA	[Long et al., 2007]	c.617-618del
c.624dupT	p.(Glu209*)	fs	POH	[Lebrun et al., 2010]	c.623-624insT
c.624dupT	p.(Glu209*)	fs	PHPIA	[Fernandez-Rebollo et al., 2013]	c.624_625insT (p.E208X) [d]
c.637C>T	p.(Gln213*)	ns	PHPIA	[Balavoine et al., 2008]	Q213X
c.640_643delGTGG	p.(Val214Thrfs*27)	fs	AHO	[Miric et al., 1993]	codon 214, delGTGG
**Intron 8**					
c.659+1G>A	p.(?)	sp	PHPIA	[Jin et al., 2011]	c.659+1G>A
c.659+2T>G	p.(?)	sp	POH	[Elli et al., 2013a]	c.662+2T>G
**Exon 9**					
c.679dupC	p.(Gln227Profs*4)	fs	POH	[Shore et al., 2002]	679-680insC
c.682C>T	p.(Arg228Cys)	ms	PHPIA	[Tam et al., 2014]	c.682C>T
c.691C>T	p.(Arg231Cys)	ms	PPHP	[Ahrens et al., 2001]	codon 231 (CGC→TGC)
c.691C>T	p.(Arg231Cys)	ms	AHO	[Weinstein et al., 2001]	Arg231Cys
c.691C>T	p.(Arg231Cys)	ms	PHPIA	[Freson et al., 2008a]	R231C
c.691C>T	p.(Arg231Cys)	ms	PPHP	[Freson et al., 2008b]	R231C
c.691C>T	p.(Arg231Cys)	ms	PHPIA	[Elli et al., 2013b]	c.694C>T
c.692G>A	p.(Arg231His)	ms	PHPIA	[Farfel et al., 1996]	R231H
c.692G>A	p.(Arg231His)	ms	PHPIA	[Ishikawa et al., 2001]	R231H (CGC→CAC)
c.692G>A	p.(Arg231His)	ms	PHPIA	[Ngai et al., 2010]	c.692G>A
**Intron 9**					
c.719-2A>G	p.(?)	sp	PHPIA	[Linglart et al., 2002]	A→G (intron 9 acceptor sp)
**Exon 10**					
c.725C>T	p.(Thr242Ile)	ms	PHPIA, PPHP	[Rickard et al., 2003]	c.725C>T
c.725C>T	p.(Thr242Ile)	ms	PHPIA	[Elli et al., 2013b]	c.728C>T
c.725delC	p.(Thr242Metfs*29)	fs	POH	[Shore et al., 2002]	725delC
c.730A>T	p.(Ile244Phe)	ms	PHPIA, PPHP	[Long et al., 2007]	c.730A>T, I224F
c.737T>C	p.(Phe246Ser)	ms	PPHP	[Rickard et al., 2003]	c.737T>C
c.739G>C	p.(Val247Leu)	ms	PHPIA	[Elli et al., 2013b]	c.742G>C
c.750C>G	p.(Ser250Arg)	ms	PHPIA	[Warner et al., 1997]	S250R (AGG→AGC)
c.750C>G	p.(Ser250Arg)	ms	PHPIA	[Long et al., 2007]	c.750C>G
c.751dupA	p.(Ser251Lysfs*49)	fs	PHPIA, PPHP	[Lado-Abeal et al., 2011]	c.750-751insA
c.772C>T	p.(Arg258Trp)	ms	PPHP	[Warner et al., 1998]	R258W (TGG→CGG)
c.772C>T	p.(Arg258Trp)	ms	PPHP	[Long et al., 2007]	c.772C>T, R258N
c.776A>T	p.(Glu259Val)	ms	PHPIA	[Ahmed et al., 1998]	codon 259 (GAG→GTG)
c.776A>T	p.(Glu259Val)	ms	PHPIA	[Rickard et al., 2003]	c.776A>T

Mutation[a]	Predicted Effect on Protein	Mutation Type[b]	Phenotype[c]	Reference	Original designation
n/a (insCCAG)		fs	PHPIA, PPHP	[Linglart et al., 2002]	codon 262, insCCAG
c.790A>C	p.(Asn264His)	ms	AHO	[Aldred et al., 2000b]	c.790A>C
c.791dupA	p.(Asn264Lysfs*36)	fs	PHPIA, PPHP	[de Arriba Munoz et al., 2011]	codon 264, insA
c.794G>A	p.(Arg265His)	ms	PHPIA, PPHP	[Bastida Eizaguirre et al., 2001]	c.794G>A
c.799dupC	p.(Gln267Profs*33)	fs	PHPIA	[Shapira et al., 1996]	codon 267, insC
c.802G>T	p.(Glu268*)	ns	PHPIA, PPHP	[Kacerovska et al., 2008]	c.802G>T
c.803A>G	p.(Glu268Gly)	ms	PHPIA	[Elli et al., 2013b]	c.806A>G
c.814delC	p.(Leu272Serfs*16)	fs	PHPIA, PPHP	[Weinstein et al., 1990]	codon 272, delC
c.814delC	p.(Leu272Serfs*16)	fs	PHPIA, PPHP	[Long et al., 2007]	c.814del
c.828C>G	p.(Ile276Met)	ms	PHPIA	[Fernandez-Rebollo et al., 2013]	c.828C>G
c.835_839dupAACAG	p.(Trp281Thrfs*9)	fs	POH	[Shore et al., 2002]	835-839duplAACAG
c.838A>G	p.(Arg280Gly)	ms	PHPIA	[Lim et al., 2002]	Arg280Gly
c.838A>T	p.(Arg280*)	ns	OC	[Huh et al., 2014]	c.838A>T
c.839G>A	p.(Arg280Lys)	ms	PHPIA	[Linglart et al., 2002]	R280K (G→C) [d]
c.839G>T	p.(Arg280Ile)	ms	PHPIA	[Fernandez-Rebollo et al., 2013]	c.839G>T
**Intron 10**					
c.839+1G>C	p.(?)	sp	PHPIA, PPHP	[Weinstein et al., 1990]	intron10 +1G→C
c.839+1G>C	p.(?)	sp	PHPIA, PPHP	[Long et al., 2007]	IVS10+1G>C
c.840-2A>G	p.(?)	sp	PHPIA, PPHP	[Wu, Y. L. et al., 2014]	c.840-2A>G
**Exon 11**					
c.841T>C	p.(Trp281Arg)	ms	POH	[Chan et al., 2004]	841T>C
c.845T>C	p.(Leu282Pro)	ms	AHO	[Aldred et al., 2000b]	c.845T>C
c.860_861delTG	p.(Val287Aspfs*12)	fs	PHPIA	[Mantovani et al., 2000]	codon 287, delTG
c.860_861delTG	p.(Val287Aspfs*12)	fs	POH	[Faust et al., 2003]	860-861delTG
c.860_861delTG	p.(Val287Aspfs*12)	fs	PHPIA	[de Sanctis et al., 2004]	c.861-862delTG [d]
c.860_861delTG	p.(Val287Aspfs*12)	fs	PHPIA	[Elli et al., 2013b]	c.863_864del
c.871delC	p.(Leu291Serfs*44)	fs	PHPIA	[Sun et al., 2009]	codon 291 del1bp
c.892G>C	p.(Ala298Pro)	ms	PHPIA (2 kindreds)	[Ahrens et al., 2001]	codon 298 (GCT→CCT)
c.896dupA	p.(Lys300Glufs*10)	fs	PHPIA	[Ahrens et al., 2001]	codon 299, insA(GAG→GAAG)
n/a	n/a	fs	PHPIA	[Fernandez-Rebollo et al., 2013]	c.896_910del (p.E299GfsX36) [e]
c.909dupT	p.(Gly304Trpfs*6)	fs	AHO	[Aldred et al., 2000b]	c.909-910insT
c.938C>T	p.(Pro313Leu)	ms	PHPIA, PPHP	[Ahrens et al., 2001]	codon 313 (CCA→CTA)
c.952T>C	p.(Tyr318His)	ms	PHPIA, PPHP	[Miao et al., 2011]	c.952T>C
c.960_961dupTC	p.(Pro321Leufs*15)	fs	POH	[Shore et al., 2002]	960-961insCT
**Intron 11**					
c.971-1G>A	p.(?)	sp	PHPIA	[Lecumberri et al., 2009]	c.971-1G>A
**Exon 12**					
c.984delC	p.(Gly329Glufs*6)	fs	PHPIA	[Freson et al., 2008b]	codon 328, del 1bp

Mutation[a]	Predicted Effect on Protein	Mutation Type[b]	Phenotype[c]	Reference	Original designation
c.996_1018dup	p.(Phe340Tyrfs*3)	fs	PHPIA	[Elli et al., 2013b]	c.1021_1022insACG CGTGACCCGGGC CAAGTACT
c.1006C>T	p.(Arg336Trp)	ms	PHPIA	[Ahrens et al., 2001]	codon 336 (CGG→TGG)
c.1006C>T	p.(Arg336Trp)	ms	PHPIA	[De Sanctis et al., 2003]	c.1006C>T
c.1006C>T	p.(Arg336Trp)	ms	PHPIA (2 kindreds)	[Elli et al., 2013b]	c.1009C>T
c.1006dupC	p.(Arg336Profs*9)	fs	POH, PHPIA	[Long et al., 2007]	c.1007insC
c.1014G>C	p.(Lys338Asn)	ms	PHPIA, PPHP	[Pohlenz et al., 2003]	L338N (AAG→AAC)
c.1021_1024delATTC	p.(Ile341Glufs*5)	fs	PHPIA	[Elli et al., 2013b]	c.1021_1024del
c.1024C>T	p.(Arg342*)	ns	PHPIA	[Linglart et al., 2002]	R342X
c.1024C>T	p.(Arg342*)	ns	POH	[Goto et al., 2010]	R342X
c.1027_1028delGA	p.(Asp343*)	fs	PHPIA, PPHP	[Wu, Y. L. et al., 2014]	c.1027_1028delGA
**Intron 12**					
c.1039-1G>A	p.(?)	sp	POH, PPHP, PHPIA	[Lebrun et al., 2010]	c.1039-1G>A
c.1039-1G>C	p.(?)	sp	POH	[Shore et al., 2002]	IVS12 -1G→C
**Exon 13**					
c.1053_1077dup	p.(Tyr360Glnfs*19)	fs	POH	[Shore et al., 2002]	1053-1077dupl(25n)
c.1070A>T	p.(His357Leu)	ms	PPHP	[Ahrens et al., 2001]	codon 357 (CAC→CTC)
c.1082dupC	p.(His362Serfs*9)	fs	PHPIA	[Germain-Lee et al., 2003]	1083insC
c.1082dupC	p.(His362Serfs*9)	fs	PHPIA	[Long et al., 2007]	c.1083insC
c.1085A>C	p.(His362Pro)	ms	PHPIA	[Long et al., 2007]	c.1088A>C, H362P [d]
c.1085A>C	p.(His362Pro)	ms	PHPIA	[Linglart et al., 2006]	c.1085A→C
c.1096G>T	p.(Ala366Ser)	ms	PHPIA with testotoxicosis (2 kindreds)	[Iiri et al., 1994]	A366S
c.1097_1108dup	p.(Ala366_Thr369dup)	if	PHPIA	[Aldred et al., 2000a]	c.1107-1108ins GCTGTGGACACT
c.1100_1101insA	p.(Asp368Glyfs*3)	fs	PHPIA, PPHP	[Lubell et al., 2009]	c.1100-1101insA
c.1106_1107delCT	p.(Thr369Argfs*12)	fs	AHO	[Aldred et al., 2000b]	c.1106-1107delCT
c.1107_1108delTG	p.(Asn371Hisfs*10)	fs	AHO	[Aldred et al., 2000b]	c.1107-1108delTG
c.1107_1108delTG	p.(Asn371Hisfs*10)	fs	POH	[Schimmel et al., 2010]	c.1107-1108delTG
c.1107_1108delTG	p.(Asn371Hisfs*10)	fs	PPHP	[Elli et al., 2013b]	c.1110_1111del
c.1129_1131delAAC	p.(Asn377del)	if	PHPIA	[Ahrens et al., 2001]	codon 317 (delAAC)
c.1129_1131delAAC	p.(Asn377del)	if	PHPIA	[Graul-Neumann et al., 2009]	c.1129-1131delAAC
c.1146_1148delCAT	p.(Ile383del)	if	PHPIB	[Wu, W. I. et al., 2001]	codon 382, delCAT
n/a	p.(Ile383del)	if	AHO	[Ringel et al., 1996]	del Ile383
c.1150C>T	p.(Gln384*)	ns	PHPIA	[Garavelli et al., 2005]	codon 384 (CAG→TAG)
c.1150C>T	p.(Gln384*)	ns	PHPIA	[Elli et al., 2013b]	c.1153C>T
c.1154G>A	p.(Arg385His)	ms	PHPIA	[Schwindinger et al., 1994]	R385H (G→A)
c.1154G>A	p.(Arg385His)	ms	PHPIA	[Tamada et al., 2008]	R385H
c.1163T>C	p.(Leu388Pro)	ms	PHPIA, PPHP	[Thiele et al., 2011]	c.1163T>C
c.1163T>G	p.(Leu388Arg)	ms	PHPIC, PPHP	[Thiele et al., 2011]	c.1163T>G
c.1173C>A or G (not specified)	p.(Tyr391*)	ns	PHPIC	[Linglart et al., 2002]	Y391X
c.1174G>A	p.(Glu392Lys)	ms	PHPIA	[Germain-Lee et al., 2003]	1174G>A
c.1174G>A	p.(Glu392Lys)	ms	PHPIA	[Long et al., 2007]	c.1174G>A

**Mutation**[a]	**Predicted Effect on Protein**	**Mutation Type**[b]	**Phenotype**[c]	**Reference**	**Original designation**
c.1174G>A	p.(Glu392Lys)	ms	PHPIC, PPHP	[Thiele et al., 2011]	c.1174G>A
c.1174G>A	p.(Glu392Lys)	ms	PPHP	[Wu, Y. L. et al., 2014]	c.1174G>A
c.1174G>T	p.(Glu392*)	ns	PHPIC, PPHP (2 kindreds)[f]	[De Sanctis et al., 2003] [Thiele et al., 2011]	c.1174G>T
Gross deletion	p.0?	gross deletion	PHPIA	[Fernandez-Rebollo et al., 2010]	30Kb deletion (exons A/B→intron 5)
Gross deletion	p.0?	gross deletion	PHPIA, PPHP	[Mitsui et al., 2012]	850Kb deletion
Gross deletion	p.0?	gross deletion	PHPIA	[Cho et al., 2013]	deletion exons 1-13

[a]Mutations are numbered in relation to the *GNAS* cDNA reference sequence (GenBank accession number NM_000516.4), whereby nucleotide +1 corresponds to the A of the ATG-translation initiation codon.
[b]Mutation type: fs, frameshift mutation; ns, nonsense mutation; ms, missense mutation; if, in-frame deletion or insertion; sp, splice site mutation.
[c]Phenotype: PHPIA, pseudohypoparathyroidism type IA; PPHP, pseudopseudohypoparathyroidism; AHO, Albright Hereditary Osteodystrophy (when not specified as PHPIA or PPHP); PHPIB, pseudohypoparathyroidism type IB; PHPIC, pseudohypoparathyroidism type IC; POH, progressive osseous heteroplasia; OC, osteoma cutis (isolated). The number of reported kindreds is included (when more than one).
[d]Discordant codon/nucleotide number in the original report.
[e]Ambiguity between nucleotide number and type of mutation (in frame deletion vs frameshift).
[f]Previously classified as PHPIA.
[g]Not determined if both mutations are on the same allele.
n/a, exact nucleotide change not available in the original report.
[§] Variant submitted to a locus-specific public database (www.lovd.nl/GNAS).

Supp. References

Ahmed SF, Dixon PH, Bonthron DT, Stirling HF, Barr DG, Kelnar CJ, Thakker RV. 1998. GNAS1 mutational analysis in pseudohypoparathyroidism. *Clin Endocrinol (Oxf)* **49**(4): 525-531.

Ahrens W, Hiort O, Staedt P, Kirschner T, Marschke C, Kruse K. 2001. Analysis of the GNAS1 gene in Albright's hereditary osteodystrophy. *J Clin Endocrinol Metab* **86**(10): 4630-4634.

Aldred MA, Bagshaw RJ, Macdermot K, Casson D, Murch SH, Walker-Smith JA, Trembath RC. 2000a. Germline mosaicism for a GNAS1 mutation and Albright hereditary osteodystrophy. *J Med Genet* **37**(11): E35.

Aldred MA, Trembath RC. 2000b. Activating and inactivating mutations in the human GNAS1 gene. *Hum Mutat* **16**(3): 183-189.

Alvarez F, Kottler ML, Paul C, Gennero I, Salles JP, Mazereeuw-Hautier J. 2010. Albright hereditary osteodystrophy: report of a particular clinical phenotype caused by a novel GNAS mutation. *J Eur Acad Dermatol Venereol* **24**(8): 974-975.

Alves C, Sampaio S, Barbieri AM, Mantovani G. 2013. Pseudohypoparathyroidism type Ia: a novel GNAS mutation in a Brazilian boy presenting with an early primary hypothyroidism. *J Pediatr Endocrinol Metab*: 1-4.

Balavoine AS, Ladsous M, Velayoudom FL, Vlaeminck V, Cardot-Bauters C, d'Herbomez M, Wemeau JL. 2008. Hypothyroidism in patients with pseudohypoparathyroidism type Ia: clinical evidence of resistance to TSH and TRH. *Eur J Endocrinol* **159**(4): 431-437.

Bastida Eizaguirre M, Iturbe Ortiz De Urbina R, Arto Urzainqui M, Ezquerra Larreina R, Escalada San Martin J. 2001. [Albright hereditary osteodystrophy: identification of a novel mutation in a family]. *An Esp Pediatr* **54**(6): 598-600.

Chan I, Hamada T, Hardman C, McGrath JA, Child FJ. 2004. Progressive osseous heteroplasia resulting from a new mutation in the GNAS1 gene. *Clin Exp Dermatol* **29**(1): 77-80.

Cho SY, Yoon YA, Ki CS, Huh HJ, Yoo HW, Lee BH, Kim GH, Yoo JH, Kim SY, Kim SJ, Sohn YB, Park SW, et al. 2013. Clinical characterization and molecular classification of 12 Korean patients with pseudohypoparathyroidism and pseudopseudohypoparathyroidism. *Exp Clin Endocrinol Diabetes* **121**(9): 539-545.

de Arriba Munoz A, Labarta Aizpun JI, Dehesa EM, Calvo MT, Perez de Nanclares G, Ferrandez-Longas A. 2011. [Different expression of the Asn264LysfsX35 mutation of the GNAS gene in a family with pseudohypoparathyroidism.]. *An Pediatr (Barc)* **74**(2): 116-121.

De Sanctis L, Romagnolo D, Olivero M, Buzi F, Maghnie M, Scire G, Crino A, Baroncelli GI, Salerno M, Di Maio S, Cappa M, Grosso S, et al. 2003. Molecular analysis of the GNAS1 gene for the correct diagnosis of Albright hereditary osteodystrophy and pseudohypoparathyroidism. *Pediatr Res* **53**(5): 749-755.

de Sanctis L, Vai S, Andreo MR, Romagnolo D, Silvestro L, de Sanctis C. 2004. Brachydactyly in 14 genetically characterized pseudohypoparathyroidism type Ia patients. *J Clin Endocrinol Metab* **89**(4): 1650-1655.

Dekelbab BH, Aughton DJ, Levine MA. 2009. Pseudohypoparathyroidism type 1A and morbid obesity in infancy. *Endocr Pract* **15**(3): 249-253.

Eddy MC, Jan De Beur SM, Yandow SM, McAlister WH, Shore EM, Kaplan FS, Whyte MP, Levine MA. 2000. Deficiency of the alpha-subunit of the stimulatory G protein and severe extraskeletal ossification. *J Bone Miner Res* **15**(11): 2074-2083.

Elli FM, Barbieri AM, Bordogna P, Ferrari P, Bufo R, Ferrante E, Giardino E, Beck-Peccoz P, Spada A, Mantovani G. 2013a. Screening for GNAS genetic and epigenetic alterations in progressive osseous heteroplasia: First Italian series. *Bone* **56**(2): 276-280.

Elli FM, deSanctis L, Ceoloni B, Barbieri AM, Bordogna P, Beck-Peccoz P, Spada A, Mantovani G. 2013b. Pseudohypoparathyroidism type Ia and pseudo-pseudohypoparathyroidism: the growing spectrum of GNAS inactivating mutations. *Hum Mutat* **34**(3): 411-416.

Farfel Z, Iiri T, Shapira H, Roitman A, Mouallem M, Bourne HR. 1996. Pseudohypoparathyroidism, a novel mutation in the betagamma-contact region of Gsalpha impairs receptor stimulation. *J Biol Chem* **271**(33): 19653-19655.

Faust RA, Shore EM, Stevens CE, Xu M, Shah S, Phillips CD, Kaplan FS. 2003. Progressive osseous heteroplasia in the face of a child. *Am J Med Genet A* **118A**(1): 71-75.

Fernandez-Rebollo E, Barrio R, Perez-Nanclares G, Carcavilla A, Garin I, Castano L, de Nanclares GP. 2008. New mutation type in pseudohypoparathyroidism type Ia. *Clin Endocrinol (Oxf)* **69**(5): 705-712.

Fernandez-Rebollo E, Garcia-Cuartero B, Garin I, Largo C, Martinez F, Garcia-Lacalle C, Castano L, Bastepe M, Perez de Nanclares G. 2010. Intragenic GNAS deletion involving exon A/B in pseudohypoparathyroidism type 1A resulting in an apparent loss of exon A/B methylation: potential for misdiagnosis of pseudohypoparathyroidism type 1B. *J Clin Endocrinol Metab* **95**(2): 765-771.

Fernandez-Rebollo E, Lecumberri B, Gaztambide S, Martinez-Indart L, Perez de Nanclares G, Castano L. 2013. Endocrine profile and phenotype-(epi)genotype correlation in Spanish patients with pseudohypoparathyroidism. *J Clin Endocrinol Metab* **98**(5): E996-1006.

Fischer JA, Egert F, Werder E, Born W. 1998. An inherited mutation associated with functional deficiency of the alpha-subunit of the guanine nucleotide-binding protein Gs in pseudo- and pseudopseudohypoparathyroidism. *J Clin Endocrinol Metab* **83**(3): 935-938.

Freson K, Izzi B, Jaeken J, Van Helvoirt M, Thys C, Wittevrongel C, de Zegher F, Van Geet C. 2008a. Compound heterozygous mutations in the GNAS gene of a boy with morbid obesity, thyroid-stimulating hormone resistance, pseudohypoparathyroidism, and a prothrombotic state. *J Clin Endocrinol Metab* **93**(12): 4844-4849.

Freson K, Izzi B, Labarque V, Van Helvoirt M, Thys C, Wittevrongel C, Bex M, Bouillon R, Godefroid N, Proesmans W, de Zegher F, Jaeken J, et al. 2008b. GNAS defects identified by stimulatory G protein alpha-subunit signalling studies in platelets. *J Clin Endocrinol Metab* **93**(12): 4851-4859.

Garavelli L, Pedori S, Zanacca C, Caselli G, Loiodice A, Mantovani G, Ammenti A, Virdis R, Banchini G. 2005. Albright's hereditary osteodystrophy (pseudohypoparathyroidism type Ia): clinical case with a novel mutation of GNAS1. *Acta Biomed* **76**(1): 45-48.

Gelfand IM, Hub RS, Shore EM, Kaplan FS, Dimeglio LA. 2007. Progressive osseous heteroplasia-like heterotopic ossification in a male infant with pseudohypoparathyroidism type Ia: a case report. *Bone* **40**(5): 1425-1428.

Germain-Lee EL, Groman J, Crane JL, Jan de Beur SM, Levine MA. 2003. Growth hormone deficiency in pseudohypoparathyroidism type 1a: another manifestation of multihormone resistance. *J Clin Endocrinol Metab* **88**(9): 4059-4069.

Goto M, Mabe H, Nishimura G, Katsumata N. 2010. Progressive osseous heteroplasia caused by a novel nonsense mutation in the GNAS1 gene. *J Pediatr Endocrinol Metab* **23**(3): 303-309.

Graul-Neumann LM, Bach A, Albani M, Ringe H, Weimann A, Kress W, Hiort O, Bartsch O. 2009. Boy with pseudohypoparathyroidism type 1a caused by GNAS gene mutation (deltaN377), Crouzon-like craniosynostosis, and severe trauma-induced bleeding. *Am J Med Genet A* **149A**(7): 1487-1493.

Huh JY, Kwon MJ, Seo KY, Kim MK, Chae KY, Kim SH, Ki CS, Yoon MS, Kim DH. 2014. Novel nonsense GNAS mutation in a 14-month-old boy with plate-like osteoma cutis and medulloblastoma. *J Dermatol*.

Iiri T, Herzmark P, Nakamoto JM, van Dop C, Bourne HR. 1994. Rapid GDP release from Gs alpha in patients with gain and loss of endocrine function. *Nature* **371**(6493): 164-168.

Inta IM, Choukair D, Bender S, Kneppo C, Knauer-Fischer S, Meyenburg K, Ivandic B, Pfister SM, Bettendorf M. 2014. Guanine Nucleotide-Binding Protein alpha Subunit Hypofunction in Children with Short Stature and Disproportionate Shortening of the 4th and 5th Metacarpals. *Horm Res Paediatr*.

Ishikawa Y, Tajima T, Nakae J, Nagashima T, Satoh K, Okuhara K, Fujieda K. 2001. Two mutations of the Gsalpha gene in two Japanese patients with sporadic pseudohypoparathyroidism type Ia. *J Hum Genet* **46**(7): 426-430.

Jin HY, Lee BH, Choi JH, Kim GH, Kim JK, Lee JH, Yu J, Yoo JH, Ko CW, Lim HH, Chung HR, Yoo HW. 2011. Clinical characterization and identification of two novel mutations of the GNAS gene in patients with pseudohypoparathyroidism and pseudopseudohypoparathyroidism. *Clin Endocrinol (Oxf)* **75**(2): 207-213.

Kacerovska D, Nemcova J, Pomahacova R, Michal M, Kazakov DV. 2008. Cutaneous and superficial soft tissue lesions associated with Albright hereditary osteodystrophy: clinicopathological and molecular genetic study of 4 cases, including a novel mutation of the GNAS gene. *Am J Dermatopathol* **30**(5): 417-424.

Klaassens M, Blom EW, Schrander JJ, Ris-Stalpers C, Nieuwenhuijzen Kruseman AC, van Steensel MA, Schrander-Stumpel CT. 2010. Unique skin changes in a case of Albright hereditary osteodystrophy caused by a rare GNAS1 mutation. *Br J Dermatol* **162**(3): 690-694.

Klagge A, Jessnitzer B, Pfaeffle R, Stumvoll M, Fuhrer D. 2010. A novel GNAS1 mutation in a German family with Albright's hereditary osteodystrophy. *Exp Clin Endocrinol Diabetes* **118**(9): 586-590.

Lado-Abeal J, Castro-Piedras I, Palos-Paz F, Labarta-Aizpun JI, Albero-Gamboa R. 2011. A family with congenital hypothyroidism caused by a combination of loss-of-function mutations in the thyrotropin receptor and adenylate cyclase-stimulating G alpha-protein subunit genes. *Thyroid* **21**(2): 103-109.

Lebrun M, Richard N, Abeguile G, David A, Coeslier Dieux A, Journel H, Lacombe D, Pinto G, Odent S, Salles JP, Taieb A, Gandon-Laloum S, et al. 2010. Progressive osseous heteroplasia: a model for the imprinting effects of GNAS inactivating mutations in humans. *J Clin Endocrinol Metab* **95**(6): 3028-3038.

Lecumberri B, Fernandez-Rebollo E, Sentchordi L, Saavedra P, Bernal-Chico A, Pallardo LF, Jimenez Bustos JM, Castano L, De Santiago M, Hiort O, Perez de Nanclares G, Bastepe M. 2009. Coexistence of two different pseudohypoparathyroidism subtypes (Ia and Ib) in the same kindred with independent Gs{alpha} coding mutations and GNAS imprinting defects. *J Med Genet* **47**(4): 276-280.

Lietman SA, Goldfarb J, Desai N, Levine MA. 2008. Preimplantation genetic diagnosis for severe albright hereditary osteodystrophy. *J Clin Endocrinol Metab* **93**(3): 901-904.

Lim SH, Poh LK, Cowell CT, Tey BH, Loke KY. 2002. Mutational analysis of the GNAS1 exons encoding the stimulatory G protein in five patients with pseudohypoparathyroidism type 1a. *J Pediatr Endocrinol Metab* **15**(3): 259-268.

Linglart A, Carel JC, Garabedian M, Le T, Mallet E, Kottler ML. 2002. GNAS1 lesions in pseudohypoparathyroidism Ia and Ic: genotype phenotype relationship and evidence of the maternal transmission of the hormonal resistance. *J Clin Endocrinol Metab* **87**(1): 189-197.

Linglart A, Mahon MJ, Kerachian MA, Berlach DM, Hendy GN, Juppner H, Bastepe M. 2006. Coding GNAS mutations leading to hormone resistance impair in vitro agonist- and cholera toxin-induced adenosine cyclic 3',5'-monophosphate formation mediated by human XLalphas. *Endocrinology* **147**(5): 2253-2262.

Long DN, McGuire S, Levine MA, Weinstein LS, Germain-Lee EL. 2007. Body mass index differences in pseudohypoparathyroidism type 1a versus pseudopseudohypoparathyroidism may implicate

paternal imprinting of Galpha(s) in the development of human obesity. *J Clin Endocrinol Metab* **92**(3): 1073-1079.

Lubell T, Garzon M, Anyane Yeboa K, Shah B. 2009. A Novel Mutation Causing Pseudohypoparathyroidism 1A with Congenital Hypothyroidism and Osteoma Cutis. *J Clin Res Pediatr Endocrinol* **1**(5): 244-247.

Luttikhuis ME, Wilson LC, Leonard JV, Trembath RC. 1994. Characterization of a de novo 43-bp deletion of the Gs alpha gene (GNAS1) in Albright hereditary osteodystrophy. *Genomics* **21**(2): 455-457.

Mantovani G, Romoli R, Weber G, Brunelli V, De Menis E, Beccio S, Beck-Peccoz P, Spada A. 2000. Mutational analysis of GNAS1 in patients with pseudohypoparathyroidism: identification of two novel mutations. *J Clin Endocrinol Metab* **85**(11): 4243-4248.

Miao ZM, Wang C, Wang BB, Meng DM, Su DM, Cheng Z, Wen QL, Han L, Yu Q, Ma X, Li CG. 2011. Identification of a novel mutation in a pseudohypoparathyroidism family. *Int J Endocrinol* **2011**: 509549.

Miric A, Vechio JD, Levine MA. 1993. Heterogeneous mutations in the gene encoding the alpha-subunit of the stimulatory G protein of adenylyl cyclase in Albright hereditary osteodystrophy. *J Clin Endocrinol Metab* **76**(6): 1560-1568.

Mitsui T, Nagasaki K, Takagi M, Narumi S, Ishii T, Hasegawa T. 2012. A family of pseudohypoparathyroidism type Ia with an 850-kb submicroscopic deletion encompassing the whole GNAS locus. *Am J Med Genet A* **158A**(1): 261-264.

Mouallem M, Shaharabany M, Weintrob N, Shalitin S, Nagelberg N, Shapira H, Zadik Z, Farfel Z. 2008. Cognitive impairment is prevalent in pseudohypoparathyroidism type Ia, but not in pseudopseudohypoparathyroidism: possible cerebral imprinting of Gsalpha. *Clin Endocrinol (Oxf)* **68**(2): 233-239.

Nakamoto JM, Sandstrom AT, Brickman AS, Christenson RA, Van Dop C. 1998. Pseudohypoparathyroidism type Ia from maternal but not paternal transmission of a Gsalpha gene mutation. *Am J Med Genet* **77**(4): 261-267.

Ngai YF, Chijiwa C, Mercimek-Mahmutoglu S, Stewart L, Yong SL, Robinson WP, Gibson WT. 2010. Pseudohypoparathyroidism type 1a and the GNAS p.R231H mutation: Somatic mosaicism in a mother with two affected sons. *Am J Med Genet A* **152A**(11): 2784-2790.

Nwosu BU, Lee MM. 2009. Pseudohypoparathyroidism type 1a and insulin resistance in a child. *Nat Rev Endocrinol* **5**(6): 345-350.

Park CH, Park HD, Lee SY, Kim JW, Sohn YB, Park SW, Jin DK. 2010. Clinical, biochemical, and genetic analysis of korean patients with pseudohypoparathyroidism type Ia. *Ann Clin Lab Sci* **40**(3): 261-266.

Patten JL, Johns DR, Valle D, Eil C, Gruppuso PA, Steele G, Smallwood PM, Levine MA. 1990. Mutation in the gene encoding the stimulatory G protein of adenylate cyclase in Albright's hereditary osteodystrophy. *N Engl J Med* **322**(20): 1412-1419.

Pinsker JE, Rogers W, McLean S, Schaefer FV, Fenton C. 2006. Pseudohypoparathyroidism type 1a with congenital hypothyroidism. *J Pediatr Endocrinol Metab* **19**(8): 1049-1052.

Pohlenz J, Ahrens W, Hiort O. 2003. A new heterozygous mutation (L338N) in the human Gsalpha (GNAS1) gene as a cause for congenital hypothyroidism in Albright's hereditary osteodystrophy. *Eur J Endocrinol* **148**(4): 463-468.

Puzhko S, Goodyer CG, Kerachian MA, Canaff L, Misra M, Juppner H, Bastepe M, Hendy GN. 2011. Parathyroid hormone signaling via Galphas is selectively inhibited by an NH(2)-terminally truncated Galphas: implications for pseudohypoparathyroidism. *J Bone Miner Res* **26**(10): 2473-2485.

Reis MT, Cattani A, Mendonca BB, Correa PH, Martin RM. 2010. A novel GNAS mutation in an infant boy with pseudohypoparathyroidism type Ia and normal serum calcium and phosphate levels. *Arq Bras Endocrinol Metabol* **54**(8): 728-731.

Rickard SJ, Wilson LC. 2003. Analysis of GNAS1 and overlapping transcripts identifies the parental origin of mutations in patients with sporadic Albright hereditary osteodystrophy and reveals a model system in which to observe the effects of splicing mutations on translated and untranslated messenger RNA. *Am J Hum Genet* **72**(4): 961-974.

Riepe FG, Ahrens W, Krone N, Folster-Holst R, Brasch J, Sippell WG, Hiort O, Partsch CJ. 2005. Early manifestation of calcinosis cutis in pseudohypoparathyroidism type Ia associated with a novel mutation in the GNAS gene. *Eur J Endocrinol* **152**(4): 515-519.

Ringel MD, Schwindinger WF, Levine MA. 1996. Clinical implications of genetic defects in G proteins. The molecular basis of McCune-Albright syndrome and Albright hereditary osteodystrophy. *Medicine (Baltimore)* **75**(4): 171-184.

Rump P, Jongbloed JD, Sikkema-Raddatz B, Mundlos S, Klopocki E, van der Luijt RB. 2011. Madelung deformity in a girl with a novel and de novo mutation in the GNAS gene. *Am J Med Genet A* **155A**(10): 2566-2570.

Savas Erdeve S, Berberoglu M, Siklar Z, Evliyaoglu O, Hiort O, Ocal G. 2010. Long-term follow-up of a pseudohypoparathyroidism type 1A patient with missense mutation (Pro115Ser) in exon 5. *J Clin Res Pediatr Endocrinol* **2**(2): 85-88.

Schimmel RJ, Pasmans SG, Xu M, Stadhouders-Keet SA, Shore EM, Kaplan FS, Wulffraat NM. 2010. GNAS-associated disorders of cutaneous ossification: two different clinical presentations. *Bone* **46**(3): 868-872.

Schrander DE, Welting TJ, Caron MM, Schrander JJ, van Rhijn LW, Korver-Keularts I, Schrander-Stumpel CT. 2014. Endochondral ossification in a case of progressive osseous heteroplasia in a young female child. *J Pediatr Orthop B*.

Schwindinger WF, Miric A, Zimmerman D, Levine MA. 1994. A novel Gs alpha mutant in a patient with Albright hereditary osteodystrophy uncouples cell surface receptors from adenylyl cyclase. *J Biol Chem* **269**(41): 25387-25391.

Semiz S, Duzcan F, Candemir M, Caner V, Thiele S, Semiz E, Hiort O. 2009. Pseudohypoparathyroidism type IA (PHP-Ia): maternally inherited GNAS gene mutation. *J Pediatr Endocrinol Metab* **22**(2): 107-108.

Shapira H, Mouallem M, Shapiro MS, Weisman Y, Farfel Z. 1996. Pseudohypoparathyroidism type Ia: two new heterozygous frameshift mutations in exons 5 and 10 of the Gs alpha gene. *Hum Genet* **97**(1): 73-75.

Shore EM, Ahn J, Jan de Beur S, Li M, Xu M, Gardner RJ, Zasloff MA, Whyte MP, Levine MA, Kaplan FS. 2002. Paternally inherited inactivating mutations of the GNAS1 gene in progressive osseous heteroplasia. *N Engl J Med* **346**(2): 99-106.

Sun LH, Cui B, Zhao HY, Tao B, Wang WQ, Li XY, Ning G, Liu JM. 2009. Identification of a novel GNAS mutation for pseudohypoparathyroidism in a Chinese family. *Endocrine* **36**(1): 25-29.

Tam VH, Chen SP, Mak CM, Fung LM, Lee CY, Chan AY. 2014. A novel mutation in pseudohypoparathyroidism type 1a in a Chinese woman and her son with hypocalcaemia. *Hong Kong Med J* **20**(3): 258-260.

Tamada Y, Kanda S, Suzuki H, Tajima T, Nishiyama T. 2008. A pseudohypoparathyroidism type Ia patient with normocalcemia. *Endocr J* **55**(1): 169-173.

Thiele S, de Sanctis L, Werner R, Grotzinger J, Aydin C, Juppner H, Bastepe M, Hiort O. 2011. Functional characterization of GNAS mutations found in patients with pseudohypoparathyroidism type Ic defines a new subgroup of pseudohypoparathyroidism affecting selectively Gsalpha-receptor interaction. *Hum Mutat* **32**(6): 653-660.

Thiele S, Werner R, Ahrens W, Hoppe U, Marschke C, Staedt P, Hiort O. 2007. A disruptive mutation in exon 3 of the GNAS gene with albright hereditary osteodystrophy, normocalcemic pseudohypoparathyroidism, and selective long transcript variant Gsalpha-L deficiency. *J Clin Endocrinol Metab* **92**(5): 1764-1768.

Thiele S, Werner R, Ahrens W, Hubner A, Hinkel KG, Hoppner W, Igl B, Hiort O. 2010. Selective deficiency of Gsalpha and the possible role of alternative gene products of GNAS in Albright hereditary osteodystrophy and pseudohypoparathyroidism type Ia. *Exp Clin Endocrinol Diabetes* **118**(2): 127-132.

Walden U, Weissortel R, Corria Z, Yu D, Weinstein L, Kruse K, Dorr HG. 1999. Stimulatory guanine nucleotide binding protein subunit 1 mutation in two siblings with pseudohypoparathyroidism type 1a and mother with pseudopseudohypoparathyroidism. *Eur J Pediatr* **158**(3): 200-203.

Warner DR, Gejman PV, Collins RM, Weinstein LS. 1997. A novel mutation adjacent to the switch III domain of G(S alpha) in a patient with pseudohypoparathyroidism. *Mol Endocrinol* **11**(11): 1718-1727.

Warner DR, Weng G, Yu S, Matalon R, Weinstein LS. 1998. A novel mutation in the switch 3 region of Gsalpha in a patient with Albright hereditary osteodystrophy impairs GDP binding and receptor activation. *J Biol Chem* **273**(37): 23976-23983.

Weinstein LS, Gejman PV, de Mazancourt P, American N, Spiegel AM. 1992. A heterozygous 4-bp deletion mutation in the Gs alpha gene (GNAS1) in a patient with Albright hereditary osteodystrophy. *Genomics* **13**(4): 1319-1321.

Weinstein LS, Gejman PV, Friedman E, Kadowaki T, Collins RM, Gershon ES, Spiegel AM. 1990. Mutations of the Gs alpha-subunit gene in Albright hereditary osteodystrophy detected by denaturing gradient gel electrophoresis. *Proc Natl Acad Sci U S A* **87**(21): 8287-8290.

Weinstein LS, Yu S, Warner DR, Liu J. 2001. Endocrine manifestations of stimulatory G protein alpha-subunit mutations and the role of genomic imprinting. *Endocr Rev* **22**(5): 675-705.

Wilson LC, Oude Luttikhuis ME, Clayton PT, Fraser WD, Trembath RC. 1994. Parental origin of Gs alpha gene mutations in Albright's hereditary osteodystrophy. *J Med Genet* **31**(11): 835-839.

Winter J, Hiort O, Hermanns P, Thiele S, Pohlenz J. 2011. A new heterozygous mutation (D196N) in the Gs alpha gene as a cause for pseudohypoparathyroidism type IA in a boy who had gallstones. *J Pediatr Endocrinol Metab* **24**(5-6): 297-301.

Wu WI, Schwindinger WF, Aparicio LF, Levine MA. 2001. Selective resistance to parathyroid hormone caused by a novel uncoupling mutation in the carboxyl terminus of G alpha(s). A cause of pseudohypoparathyroidism type Ib. *J Biol Chem* **276**(1): 165-171.

Wu YL, Hwang DY, Hsiao HP, Ting WH, Huang CY, Tsai WY, Chen HC, Chao MC, Lo FS, Tsai JD, Yang S, Shih SL, et al. 2014. Mutations in pseudohypoparathyroidism 1a and pseudopseudohypoparathyroidism in ethnic chinese. *PLoS One* **9**(3): e90640.

Yeh GL, Mathur S, Wivel A, Li M, Gannon FH, Ulied A, Audi L, Olmstead EA, Kaplan FS, Shore EM. 2000. GNAS1 mutation and Cbfa1 misexpression in a child with severe congenital platelike osteoma cutis. *J Bone Miner Res* **15**(11): 2063-2073.

Yokoyama M, Takeda K, Iyota K, Okabayashi T, Hashimoto K. 1996. A 4-base pair deletion mutation of Gs alpha gene in a Japanese patient with pseudohypoparathyroidism. *J Endocrinol Invest* **19**(4): 236-241.

Yu D, Yu S, Schuster V, Kruse K, Clericuzio CL, Weinstein LS. 1999. Identification of two novel deletion mutations within the Gs alpha gene (GNAS1) in Albright hereditary osteodystrophy. *J Clin Endocrinol Metab* **84**(9): 3254-3259.

Yu S, Yu D, Hainline BE, Brener JL, Wilson KA, Wilson LC, Oude-Luttikhuis ME, Trembath RC, Weinstein LS. 1995. A deletion hot-spot in exon 7 of the Gs alpha gene (GNAS1) in patients with Albright hereditary osteodystrophy. *Hum Mol Genet* **4**(10): 2001-2002.
